# A Self-Supervised Deep Learning Reconstruction for Shortening the Breathhold and Acquisition Window in Cardiac Magnetic Resonance Fingerprinting

**DOI:** 10.3389/fcvm.2022.928546

**Published:** 2022-06-23

**Authors:** Jesse I. Hamilton

**Affiliations:** ^1^Department of Radiology, University of Michigan, Ann Arbor, MI, United States; ^2^Department of Biomedical Engineering, University of Michigan, Ann Arbor, MI, United States

**Keywords:** deep learning, deep image prior, cardiovascular imaging, low rank, multiparametric magnetic resonance imaging (MRI), magnetic resonance fingerprinting (MRF), T1 mapping, T2 mapping

## Abstract

The aim of this study is to shorten the breathhold and diastolic acquisition window in cardiac magnetic resonance fingerprinting (MRF) for simultaneous T_1_, T_2_, and proton spin density (M_0_) mapping to improve scan efficiency and reduce motion artifacts. To this end, a novel reconstruction was developed that combines low-rank subspace modeling with a deep image prior, termed DIP-MRF. A system of neural networks is used to generate spatial basis images and quantitative tissue property maps, with training performed using only the undersampled k-space measurements from the current scan. This approach avoids difficulties with obtaining *in vivo* MRF training data, as training is performed *de novo* for each acquisition. Calculation of the forward model during training is accelerated by using GRAPPA operator gridding to shift spiral k-space data to Cartesian grid points, and by using a neural network to rapidly generate fingerprints in place of a Bloch equation simulation. DIP-MRF was evaluated in simulations and at 1.5 T in a standardized phantom, 18 healthy subjects, and 10 patients with suspected cardiomyopathy. In addition to conventional mapping, two cardiac MRF sequences were acquired, one with a 15-heartbeat(HB) breathhold and 254 ms acquisition window, and one with a 5HB breathhold and 150 ms acquisition window. In simulations, DIP-MRF yielded decreased nRMSE compared to dictionary matching and a sparse and locally low rank (SLLR-MRF) reconstruction. Strong correlation (R^2^ > 0.999) with T_1_ and T_2_ reference values was observed in the phantom using the 5HB/150 ms scan with DIP-MRF. DIP-MRF provided better suppression of noise and aliasing artifacts *in vivo*, especially for the 5HB/150 ms scan, and lower intersubject and intrasubject variability compared to dictionary matching and SLLR-MRF. Furthermore, it yielded a better agreement between myocardial T_1_ and T_2_ from 15HB/254 ms and 5HB/150 ms MRF scans, with a bias of −9 ms for T_1_ and 2 ms for T_2_. In summary, this study introduces an extension of the deep image prior framework for cardiac MRF tissue property mapping, which does not require pre-training with *in vivo* scans, and has the potential to reduce motion artifacts by enabling a shortened breathhold and acquisition window.

## Introduction

Cardiac magnetic resonance (CMR) T_1_ and T_2_ mapping are useful for the detection of pathological changes in myocardial tissue, including acute (1) and chronic inflammation (2, 3), edema (4, 5), amyloid deposition (6), fatty infiltration (7), and infarct (8). Multiparametric methods have recently been developed to efficiently measure multiple tissue properties during one scan (9–12). Cardiac magnetic resonance fingerprinting (MRF) is one such technique that uses a time-varying pulse sequence to encode several properties in magnetization signal evolutions over time (13, 14). A time series of highly undersampled images is acquired, typically with a single image frame collected per repetition time (TR). Quantitative maps are obtained using pattern recognition, where the signal evolution (or “fingerprint”) measured at each voxel is matched to a dictionary of fingerprints simulated for different tissue property values.

While simultaneous T_1_, T_2_, and proton spin density (M_0_) mapping using cardiac MRF has been demonstrated in healthy subjects (15) and cardiomyopathy patients (16), respiratory and cardiac motion present significant challenges, even when breathholding and electrocardiogram (ECG) triggering are employed. The highly accelerated non-Cartesian sampling used in cardiac MRF introduces noise-like artifacts in the measured fingerprints, and thus many image frames are collected to enable accurate pattern recognition using the corrupted signals. Several previous studies employed a relatively long breathhold of 15 heartbeats and diastolic acquisition window of approximately 250 ms as a result (15). However, this sequence may be susceptible to motion if patients have difficulty holding their breath or have elevated heart rates. While retrospective motion correction can be used (17), an alternative strategy is to shorten the breathhold and acquisition window to avoid the need for such corrections.

Shortening the MRF acquisition will result in fewer time points in each fingerprint, which can impede accurate pattern recognition. Several classes of reconstruction methods have been developed to accelerate MRF scans, including model-based reconstructions (18, 19), low-rank subspace techniques (20–22), and deep learning (23). Deep learning methods have gained particular interest for their excellent denoising capabilities and fast computation times. While some MRF deep learning reconstructions operate on single-voxel fingerprints (23, 24), others use the fingerprints from many voxels within a spatial neighborhood to estimate the tissue properties at a target voxel (25), and thus can leverage both spatial and temporal correlations in the MRF data to reduce noise and k-space undersampling artifacts. Such a method was recently demonstrated for MRF in the brain, where a convolutional neural network (CNN) reconstruction enabled a 4-fold reduction in scan time compared to conventional dictionary matching (25) and allowed for high-resolution (submillimeter) mapping (26).

However, CNN reconstructions typically require training using *in vivo* datasets, which presents a challenge for cardiac MRF. It is difficult to collect ground truth tissue property maps in the heart due physiological motion, as a scan time of several minutes would be needed to obtain fully-sampled MRF data. Furthermore, because the MRF scan is prospectively triggered, the fingerprints depend on the subject’s cardiac rhythm (14), and thus many datasets from subjects with different cardiac rhythms (including fast or irregular rhythms commonly seen in patients) would potentially be needed for training.

Recently, a deep image prior (DIP) technique was proposed for image processing tasks that does not require pre-training with ground truth datasets (27). Taking image denoising as an example, a randomly initialized CNN learns to generate a denoised image by minimizing the mean squared error loss compared to a noise-corrupted image, with no requirements for additional training data. The network architecture is typically based on a u-net (28) and is designed so that lower spatial frequencies are recovered before higher spatial frequencies (29). Therefore, the network learns to generate natural images before recovering higher frequency noise, so that training with early stopping avoids overfitting to the noisy image. When applied to inverse problems in medical imaging, a mathematical model of the image acquisition can be incorporated in the loss function, which has been applied to computed tomography (30), positron emission tomography (31), and diffusion MRI (32).

This study introduces a self-supervised deep learning reconstruction for cardiac MRF T_1_, T_2_, and M_0_ mapping for the purpose of mitigating noise, reducing k-space undersampling artifacts, and enabling a shortened acquisition to reduce motion artifacts. The proposed method, termed DIP-MRF, combines low-rank MRF subspace modeling with the denoising capabilities of a deep image prior. A system of convolutional (u-net) and fully-connected networks is used to generate spatial basis images (i.e., images in a low-dimensional subspace derived from the MRF signal evolutions) and quantitative maps, without dictionary matching and without pre-training using *in vivo* data. For each MRF acquisition, training is performed *de novo* using only the undersampled k-space measurements from the current scan by incorporating a mathematical model of the cardiac MRF data acquisition in the loss function. DIP-MRF is shown to reduce noise and undersampling artifacts compared to conventional dictionary matching and low-rank subspace reconstructions. Furthermore, DIP-MRF is leveraged to shorten the breathhold duration from 15 to 5 heartbeats and diastolic acquisition window from 250 to 150 ms, with results shown in healthy subjects and cardiomyopathy patients, which has the potential to reduce motion artifacts.

## Materials and Methods

Previous work has shown that an MRF dictionary, denoted by *D* ∈ ℂ*^p^*×*^t^*, where *p* is the number of parameter combinations and *t* is the number of time points, can be compressed along time using a truncated singular value decomposition (SVD) that retains only the first *k* singular values (33). The temporal basis functions are denoted by *V*_*k*_ ∈ ℂ*^t^*×*^k^*, which is matrix whose columns contain the first *k* right singular vectors. A compressed dictionary, denoted by *D*_*k*_ ∈ ℂ*^p^*×*^k^*, can be obtained according to *D*_*k*_=*DV*_*k*_. Similarly, if *x* ∈ ℂ*^n^*×*^t^* denotes a time series of MRF images with *n* voxels, then multiplication by *V_k* yields a set of spatial basis images in this low-dimensional subspace, denoted by *x*_*k*_=*xV*_*k*_, where *x*_*k*_ ∈ ℂ*^n^*×*^k^*. Multiplying the spatial basis images by the complex conjugate $V_{k}^{*}$ will yield a low-rank approximation to the original MRF image series, $x\approx x_{k}\,V_{k}^{*}$. Low-rank subspace reconstructions for MRF have been proposed that iteratively remove noise and undersampling artifacts from the spatial basis images, sometimes with additional regularization terms using spatial sparsity and/or locally low rank regularization, before matching to the compressed dictionary to obtain quantitative maps (21, 22, 34, 35).

**FIGURE 1:**
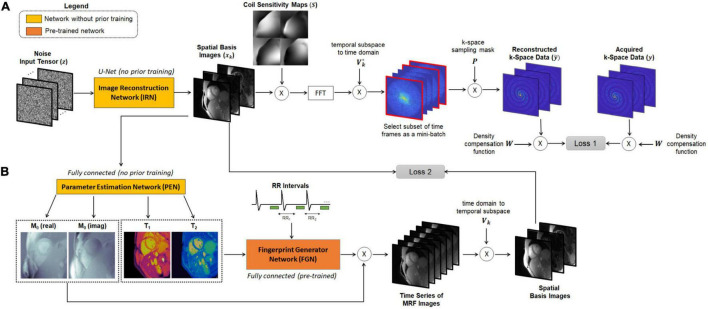
Overview of the DIP-MRF reconstruction. A system of neural networks outputs spatial basis images and T_1_, T_2_, and M_0_ maps, with no additional *in vivo* training data needed beyond the undersampled k-space data from the current scan. **(A)** The image reconstruction network (IRN) is a convolutional u-net that outputs a set of *k* spatial basis images. The input is a tensor of random numbers that remains fixed throughout training. Training is performed in a self-supervised manner by simulating the cardiac MRF forward encoding model. This step includes multiplication by coil sensitivity maps, fast Fourier transformation (FFT), projection of k-space data from the low-dimensional subspace to the time domain, and multiplication by spiral undersampling masks. The resulting k-space data are compared to the acquired k-space measurements, after density compensation, at the sampled locations using a mean squared error loss function (Loss 1), and IRN is updated using backpropagation. **(B)** A fully-connected network, referred to as the Parameter Estimation Network (PEN), uses the spatial basis images to output tissue property maps. Specifically, it outputs T_1_, T_2_, and a complex-valued M_0_ scaling term. The T_1_ map, T_2_ map, and cardiac rhythm timings (RR intervals) from the ECG are input to the fingerprint generator network, which is a pre-trained fully-connected network that can be thought of as an efficient Bloch equation simulator that rapidly outputs cardiac MRF signal evolutions (fingerprints). The simulated fingerprints at all voxels are multiplied by the complex M_0_ map to yield a time series of images. The images are projected onto the low-dimensional subspace and compared to the spatial basis images that were output by the IRN using a mean squared error loss function, and the PEN is updated using backpropagation (Loss 2). Note that the IRN and PEN are trained in parallel.

This study extends the deep image prior framework using a low-rank cardiac MRF signal model. An overview of the DIP-MRF reconstruction pipeline is shown in Figure 1. A convolutional u-net generates spatial basis images, which are input to a fully-connected network that outputs quantitative maps, neither of which require pre-training with *in vivo* data. Rather, the networks are trained in a self-supervised manner to enforce consistency with the undersampled k-space data from a single scan by incorporating the MRF forward encoding model in the loss function. The forward model includes (1) simulation of a time series of MRF images from the tissue property maps, (2) projection of images onto the low-dimensional subspace, (3) coil sensitivity encoding, and (4) spiral k-space undersampling. Calculation of the forward model is accelerated by (1) a pre-trained neural network that rapidly outputs fingerprints instead of using a more time-consuming Bloch equation simulation (36), and (2) preprocessing the spiral MRF k-space data with GRAPPA operator gridding (GROG) to obtain data in Cartesian k-space (37). The following sections will describe the DIP-MRF pipeline in more detail.

### Pre-trained Fingerprint Generator Network

**FIGURE 2:**
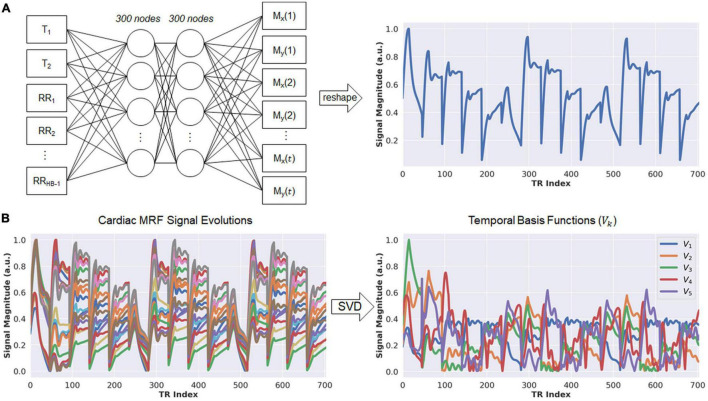
Schematic of the fingerprint generator network (FGN) and derivation of the low-dimensional subspace. **(A)** The FGN is a fully-connected network with two hidden layers. The input consists of a T_1_ value, T_2_ value, and vector of RR interval times (RR_1_, RR_2_, …, RR_*HB–1*_) recorded by the ECG, where *RR*_*i*_ denotes the elapsed time (in milliseconds) between the end of the acquisition window in heartbeat *i* and the beginning of the acquisition window in heartbeat *i+1*, and *HB* is the total number of heartbeats in the scan. The output is a vector of length *2t*, where *t* is the number of repetition times (i.e., number of time points), which contains the interleaved real and imaginary parts of an MRF fingerprint. **(B)** The FGN is used to calculate a dictionary of fingerprints for different T_1_ and T_2_ combinations specific for the patient’s cardiac rhythm timings (left panel). The SVD of the dictionary is calculated in order to derive the low-rank approximation used in the DIP-MRF forward model calculation (right panel).

Calculating the forward model requires repeated simulations of MRF signal evolutions at every iteration. To reduce computation time, this step is performed using a neural network called the Fingerprint Generator Network (FGN), which rapidly outputs signal evolutions for arbitrary T_1_, T_2_, and cardiac rhythm timings (Figure 2A) and has been described previously (36). The network is fully-connected with two hidden layers and 300 nodes per layer. The input consists of a T_1_ value, a T_2_ value, and the subject’s cardiac rhythm timings (specifically, a vector of RR interval times) recorded by the ECG during the scan. The output is a vector of length 2*t* containing interleaved real and imaginary parts of the fingerprint. The FGN is the only neural network component in the DIP-MRF pipeline that requires pre-training. The pre-training is performed only one time using fingerprints produced by a Bloch equation simulation for different T_1_, T_2_, and cardiac rhythm timings, after which the same network can be applied to any subsequent scan regardless of the subject’s cardiac rhythm. Supplementary Figure 1 gives additional details about pre-training the FGN.

### Low-Rank Signal Approximation

Although DIP-MRF does not use pattern recognition, a dictionary of fingerprints is calculated temporarily in order to derive the temporal basis functions *V_k* (33). The FGN is used to output a dictionary of approximately 23,000 fingerprints with T_1_ between 50–3,000 ms and T_2_ between 5–1,000 ms, which takes 30 ms on a GPU. Next, the SVD of the dictionary is calculated (taking approximately 1 s), and the temporal basis functions are obtained from the first *k* right singular vectors (Figure 2B). This study uses a rank of *k* = 5, which retains more than 99.9% of the energy compared to the uncompressed fingerprints.

### GRAPPA Operator Gridding Preprocessing and Coil Sensitivity Estimation

The forward model calculation requires repeated iterations between image and k-space domains. To avoid time-consuming operations using the non-uniform fast Fourier Transform (NUFFT) (38), the MRF spiral k-space data are preprocessed using GROG, a parallel imaging technique that shifts non-Cartesian k-space data to unmeasured Cartesian locations using GRAPPA weight matrices (37). The weight matrices for unit shifts along *k_x* and *k_y* are calibrated using a fully-sampled dataset; this dataset is obtained by taking the temporal average of the multicoil MRF k-space data, gridding a time-averaged image using the NUFFT, and performing an FFT to obtain multicoil Cartesian k-space data. The central 48 × 48 region of the Cartesian k-space is used for GROG calibration. Coil sensitivity maps are estimated from the time-averaged multicoil images using the adaptive combination method (39). The GROG density compensation function, denoted by *W*, is obtained by counting the number of spiral k-space points that are shifted to each Cartesian coordinate. After calibration, the GROG weights are applied to shift undersampled spiral MRF k-space data onto a Cartesian grid, and each time frame of the resulting Cartesian k-space dataset is multiplied by *W*. A binary mask, denoted by *P_i*, is stored that indicates the sampled (acquired) points on the Cartesian grid at each time index *i*.

### Neural Network Architectures

**FIGURE 3:**
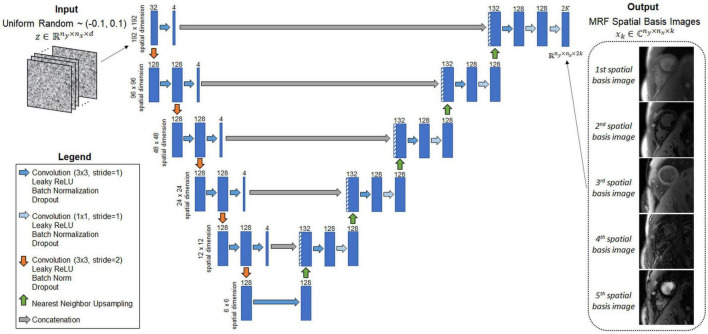
Schematic of the image reconstruction network (IRN), which outputs MRF spatial basis images. The input, *z*, is a tensor of uniformly distributed random numbers between −0.1 and 0.1 that remains fixed while training the network. The network is a u-net that performs a series of 2D convolutions. It has five downsampling and upsampling paths with multiple shortcut connections. The network outputs the MRF spatial basis images—i.e., images in a low-dimensional subspace of rank *k* that was derived from a dictionary of simulated signal evolutions, as described in Figure 2. The number of 2D filters is listed above each convolutional layer (indicated by the blue rectangles).

A convolutional u-net, which is not pre-trained, is used to output the MRF spatial basis images. This network will be called the image reconstruction network (IRN) and is shown in Figure 3. Inspired by the original DIP publication (27), the input is a tensor denoted by *z* ∈ ℝ*^n^*_*y*_×*n*_*x*_×*^d^* of uniform random numbers between −0.1 and 0.1, where *n_y* and *n_x* are the spatial dimensions in voxels, and *d* is a tunable parameter defining the number of feature channels in the first layer of the network. This study uses *d* = 32 to be consistent with the original DIP work, but this parameter was not found to have much impact on the reconstruction. The IRN performs a series of 2D convolutions followed by batch normalization, leaky ReLU activation, and an optional dropout layer. The data pass through five downsampling and upsampling paths with multiple skip connections. Downsampling is implemented using convolution with a 2 × 2 stride, and upsampling is performed using nearest neighbor interpolation followed by convolution. The network output has size *n*_*y*_×*n*_*x*_×2*k*, where the channel dimension contains the interleaved real and imaginary parts of the *k* spatial basis images.

**FIGURE 4:**
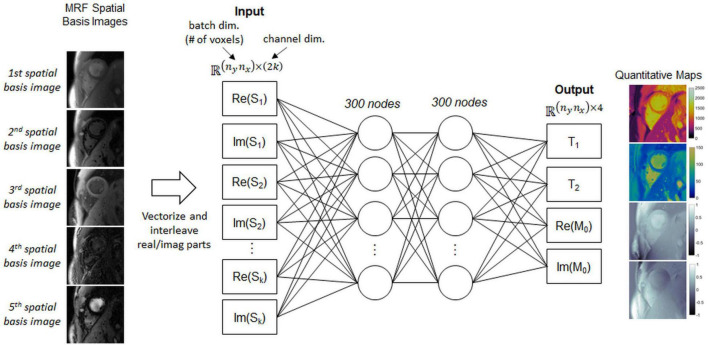
Schematic of the parameter estimation network (PEN), which estimates quantitative maps from the spatial basis images. Before being input to the network, the spatial basis images are first vectorized to have size *n_yn_x* (the batch dimension) by *2k* (the channel dimension), where the channel dimension contains interleaved real and imaginary signal intensities from the *k* spatial basis images, and *n_y* and *n_x* are the spatial dimensions (number of voxels). The network has two hidden layers with 300 nodes per layer. The output has four channels corresponding to T_1_, T_2_, and the real and imaginary parts of the M_0_ scaling term.

A fully-connected network, which also is not pre-trained, outputs quantitative T_1_, T_2_, and M_0_ maps from the spatial basis images. This network will be called the parameter estimation network (PEN) and is shown in Figure 4. The PEN has two hidden layers with 300 nodes per layer. Before being input to the network, the spatial basis images are vectorized to have size (*n*_*y*_*n*_*x*_)×(2*k*), where the second (channel) dimension contains interleaved real and imaginary signal intensities. The network output has one channel for each tissue property. As in previous MRF studies (13, 14), M_0_ is modeled as a complex-valued scaling factor between the measured and simulated fingerprints, so the output has four channels for T_1_, T_2_, and the real and imaginary parts of M_0._

### Self-Supervised Training

The IRN and PEN networks are trained *de novo* for each reconstruction in a self-supervised manner (Figure 1). Both networks are initialized with random weights and biases. Additionally, the input (*z*) to the IRN is initialized with random numbers and remains fixed throughout training. Both networks are trained in parallel using a loss function with two terms, one for updating each network. First, letting θ_**I***RN*_ denote the network parameters of the IRN, the spatial basis images generated by the IRN can be written as,


(1) $$x_{k}=\theta_{I\,R\,N}\,\left(z\right)$$

The spatial basis images are multiplied by coil sensitivity maps (*S*), transformed to k-space by performing an FFT, and multiplied by $V_{k}^{*}$ to yield time series data. To reduce memory requirements, a subset of time frames is selected as mini-batch at this point. In practice, this is implemented by using $V_{i,k}^{*}$ instead of *V_k*, where $V_{i,k}^{*}$ denotes the *i^th^* column vector from $V_{k}^{*}$ (note that multiplication by $V_{i,k}^{*}$ projects data from the subspace to the time domain and extracts only the *i^th^* time frame). The k-space data for time frame *i* are multiplied by the spiral undersampling mask for the corresponding time frame (*P_i*) and by the GROG density compensation function (*W*). The estimated multicoil k-space data for time frame *i*, denoted by ${\tilde{y}}_{i}$, can be written as,


(2) $${\tilde{y}}_{i}=W\,P_{i}\,\left(\left(F\,S\,x_{k}\right)\,V_{i,k}^{*}\right)$$

The first loss term is calculated as the mean squared error between ${\tilde{y}}_{i}$ and the acquired multicoil k-space measurements after density compensation, denoted by *y*_*i*_, at the sampled locations, and the IRN is updated using backpropagation.


(3) $$\min_{\theta_{I\,R\,N}}\sum {||y_{i}-{\tilde{y}}_{i}||}_{2}^{2}$$

The PEN is updated in parallel using a second loss term. The T_1_ and T_2_ maps output by the PEN, along with the subject’s RR interval times from the ECG, are input to the FGN to yield simulated fingerprints at each voxel location. These fingerprints are multiplied by the complex-valued M_0_ map to obtain a time series of images that are projected onto the subspace by multiplication with *V_k*. Letting θ_*PEN*_ and θ_*FGN*_ denote the network parameters of the PEN and FGN, respectively, the second loss term is calculated as the mean squared error between the resulting images and the spatial basis images output by the IRN:


(4) $$\min_{\theta_{P\,E\,N}}\sum {||x_{k}-\left(M_{0}\,\theta_{F\,G\,N}\,\left(T_{1},T_{2},R\,R\right)\right)\,V_{k}||}_{2}^{2}$$

For all experiments, training was performed for 30,000 iterations using an Adam optimizer with learning rate 0.001. DIP-MRF was implemented in Tensorflow (v2.8) with Keras on a GPU (NVIDIA Tesla v100 16GB). A mini-batch size of 32 image frames was used to calculate the loss for the IRN.

### Cardiac Magnetic Resonance Fingerprinting Acquisition Parameters

Data were collected using a fast imaging with steady state precession (FISP) cardiac MRF sequence with a 15-heartbeat (HB) breathhold and 254 ms ECG-triggered diastolic acquisition (15, 40). Variable flip angles (4–25°) and a constant TR/TE of 5.4/1.4 ms were employed. A total of 705 undersampled images were collected (one image per TR) with 47 images acquired every heartbeat. Magnetization preparation pulses were applied before the acquisition window in each heartbeat according to the following schedule, which repeated three times during the scan: HB1—inversion (21 ms), HB2—no preparation, HB3—T_2_ prep (30 ms), HB4—T_2_ prep (50 ms), HB5—T_2_ prep (80 ms).

In addition, shortened MRF acquisitions were investigated having a five-heartbeat breathhold and progressively shorter acquisition windows. These were based on the same sequence structure, with the only difference being that the flip angle pattern within each heartbeat was truncated to fit within the desired scan window. An example of a flip angle series for a shortened scan is shown in Supplementary Figure 2. All data were acquired using a 48-fold undersampled spiral k-space trajectory (41) with a readout duration of 3.4 ms, matrix size of 192 × 192, field-of-view (FOV) of 300 × 300 mm^2^, and golden angle rotation of the trajectory every TR (42).

### Simulation Experiments

Simulations were performed to investigate the feasibility of shortening the breathhold and diastolic scan window in cardiac MRF. In addition to the scan with a 15HB breathhold and 254 ms acquisition window (705 total TRs), scans with a 5HB breathhold and acquisition windows of 254 ms (235 total TRs), 200 ms (185 total TRs), 150 ms (140 total TRs), 100 ms (95 total TRs), and 50 ms (45 total TRs) were simulated. The MRF data acquisition was simulated, including Bloch equation signal simulation, coil sensitivity encoding with 8-channel sensitivity maps, and spiral k-space undersampling using the NUFFT. Complex Gaussian noise was added to the k-space data having a standard deviation of 0.1% of the maximum amplitude of the direct current (DC) signal. For each sequence variant, maps were reconstructed in three ways. In the first method (*direct matching*), one undersampled image was gridded every TR using the NUFFT, followed by dot product matching with a dictionary generated by a Bloch equation simulation to obtain T_1_, T_2_, and M_0_ maps (13). In the second method (*SLLR-MRF*), a sparse and locally low rank MRF reconstruction was performed (34), which yielded a set of *k* = 5 spatial basis images that were matched to an SVD-compressed dictionary. Locally low rank regularization with an 8 × 8 patch size and *l_1*-wavelet regularization were used with regularization weights of λ_*LLR*_=0.02 and λ_*wav*_=0.005 relative to the maximum intensity in the basis images. The reconstruction was solved using non-linear conjugate gradient descent with 25 iterations. The third method (*DIP-MRF*) consisted of GROG preprocessing followed by the DIP-MRF reconstruction. The reconstructions were compared using the normalized root mean square error (nRMSE) relative to the ground truth T_1_ and T_2_ maps, computed over all non-background voxels (i.e., all voxels where the ground truth M_0_ was non-zero).

A second set of simulations evaluated the robustness of DIP-MRF to noise. For the sequence with a 5HB breathhold and 150 ms acquisition window, complex Gaussian noise was added to the k-space data having standard deviations (σ_*N*_) of 0, 0.1, 0.2, and 0.3% relative to the maximum amplitude of the DC signal. Maps were reconstructed using direct matching, SLLR-MRF, and DIP-MRF and compared in terms of nRMSE.

A third set of simulations assessed the impact of applying dropout during training (43). For the sequence with a 5HB breathhold and 150 ms acquisition window, the DIP-MRF reconstruction was repeated where different levels of dropout (0, 10, and 20%) were applied after each convolutional layer when training the IRN, and the maps were compared in terms of nRMSE.

### Phantom Experiments

Experiments were performed using the ISMRM/NIST MRI system phantom (44) on a 1.5T scanner (MAGNETOM Sola, Siemens Healthineers, Erlangen, Germany). An 8 mm slice was planned through the T_2_ layer of the phantom, which has 14 spheres spanning a range of physiological relaxation times with T_1_ 90–2,230 ms and T_2_ 10–750 ms. An artificial heart rate of 60 bpm was simulated on the scanner. Data were collected using two cardiac MRF sequences: a sequence with a 15HB breathhold and 254 ms acquisition window and a sequence with a 5HB breathhold and 150 ms acquisition window. Maps were reconstructed using direct matching, SLLR-MRF, and DIP-MRF. Data were also acquired with conventional cardiac mapping sequences using Siemens MyoMaps software (45). T_1_ maps were collected with 5(3)3 modified look-locker inversion recovery (MOLLI) (46), and T_2_ maps were collected using a 1(3)1(3)1 T_2_-prepared balanced steady state free precession (bSSFP) sequence with T_2_ prep times of 0, 25, and 55 ms (5). Conventional cardiac mapping scans used GRAPPA with an acceleration factor of 2 and 24 autocalibration lines, 6/8 partial Fourier, a flip angle of 35°, and a scan window of 209 ms. All scans were collected with a matrix size of 192 × 192 and 300 mm^2^ FOV. Reference T_1_ values were measured using an inversion recovery spin echo sequence with TR = 10 s, TE = 12 ms, and inversion times of 21, 100, 200, 400, 800, and 1,600 ms. Reference T_2_ values were measured with a single-echo spin echo sequence with TR = 10 s and echo times of 10, 20, 40, 60, 100, 150, and 200 ms. Mean relaxation times were measured within each vial and compared to reference values using linear regression and Bland-Altman analyses (47). T_2_ values above 200 ms were excluded from analysis because the cardiac MRF sequence was not designed for that regime, considering that the longest T_2_ prep time was 80 ms (for completeness, measurements in all 14 vials are reported in the Supplementary Material).

### Scans in Healthy Subjects and Patients

Eighteen healthy subjects were scanned at 1.5T after obtaining written informed consent in this IRB-approved, HIPAA-compliant study. All scans were performed during an end-expiratory breathhold at a mid-ventricular slice position. MOLLI and T_2_-prep bSSFP mapping were performed in all subjects. Data were also acquired using 15HB/254 ms and 5HB/150 ms cardiac MRF acquisitions, and maps were reconstructed using direct matching, SLLR-MRF, and DIP-MRF. To study the effects of training with dropout and to determine the optimal dropout percentage, the DIP-MRF reconstruction was repeated in three subjects with 0, 5, 10, 20, and 30% dropout applied after each convolutional layer when training the IRN. Unless otherwise states, the DIP-MRF reconstruction used dropout levels of 10 and 20% for the 15HB/254 ms and 5HB/150 ms MRF acquisitions, respectively.

In addition, data were collected in ten patients referred for a clinical CMR exam due to suspected cardiomyopathy. Native T_1_ and T_2_ maps were collected using the same protocol as in healthy subjects. Post-contrast T_1_ and T_2_ maps were acquired 15–25 min after IV injection of 0.2 mmol/kg body weight gadoteridol (ProHance, Bracco Diagnostics Inc., Princeton, NJ, United States). While post-contrast MRF scans (both 15HB/254 ms and 5HB/150 ms versions) were performed in all patients, post-contrast MOLLI and T_2_-prep bSSFP sequences were only collected in nine and three patients, respectively.

*In vivo* data were analyzed by manually segmenting the maps according to American Heart Association (AHA) guidelines (48). The mean and standard deviation for T_1_ and T_2_ were measured within each AHA segment and over all voxels in the myocardium. Similarly, T_1_ and T_2_ values were measured within the left (LV) and right ventricular (RV) blood pools after manual segmentation, taking care to avoid trabeculations and papillary muscles. Intersubject variability was quantified as the standard deviation of the mean T_1_ or T_2_ values over all subjects. Intrasubject variability was quantified by measuring the standard deviation in T_1_ or T_2_ for each subject and then calculating the mean over all subjects. T_1_ and T_2_ measurements using different reconstruction methods within the same subject were compared using a within-subjects ANOVA test with a Bonferroni *post-hoc* test for multiple comparisons, with *p* < 0.05 indicating statistical significance, as well as Bland-Altman plots. T_1_ and T_2_ measurements between healthy subjects and patients were compared using a two-sample *t*-test.

## Results

### Simulation Experiments

**FIGURE 5:**
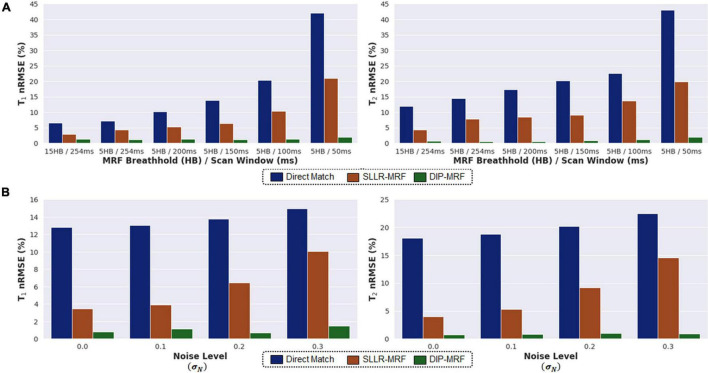
Simulation results in the MRXCAT phantom comparing the deep image prior (DIP-MRF) to direct matching and a low-rank subspace reconstruction (SLLR-MRF). **(A)** nRMSE plots of the T_1_ and T_2_ maps are shown for different cardiac MRF sequence lengths. Results are shown for MRF with a 15-heartbeat breathhold and 254 ms diastolic acquisition window, as well as shortened scans with a 5-heartbeat breathhold and successively shorter acquisition windows of 254, 200, 150, 100, and 50 ms. **(B)** nRMSE plots of the T_1_ and T_2_ maps are shown for the 5HB/150 ms MRF scan, where different amounts of random Gaussian noise having standard deviation σ_*N*_ (expressed as a percentage of the maximum DC signal level) were added to the simulated k-space data.

Figure 5A shows simulation results using MRF sequences with different breathhold and acquisition window lengths. In all cases, the nRMSE was highest with direct matching and lowest with DIP-MRF, and this difference was more pronounced for shorter sequence lengths. As the breathhold and acquisition window were shortened, nRMSE increased for direct matching and SLLR-MRF but remained consistently low for DIP-MRF. For the 15HB/254 ms sequence, the nRMSE was (T_1_ 6.5%, T_2_ 11.2%) for direct matching, (T_1_ 2.9%, T_2_ 4.3%) for SLLR-MRF, and (T_1_ 1.4%, T_2_ 0.7%) for DIP-MRF. For the 5HB/150 ms sequence, the nRMSE was (T_1_ 13.4%, T_2_ 20.2%) for direct matching, (T_1_ 6.4%, T_2_ 9.1%) for SLLR-MRF, and (T_1_ 1.2%, T_2_ 0.8%) for DIP-MRF. Supplementary Figure 3 shows examples of T_1_, T_2_, and M_0_ maps from the simulation study.

Figure 5B plots the nRMSE for the 5HB/150 ms sequence as the k-space data were corrupted with different amounts of complex Gaussian noise. The nRMSE was highest with direct matching and lowest with DIP-MRF at all noise levels. At the highest noise level tested (σ_*N*_=0.3% of the DC signal), the nRMSE was (T_1_ 14.9%, T_2_ 22.5%) for direct matching, (T_1_ 10.0%, T_2_ 14.6%) for SLLR-MRF, and (T_1_ 1.5%, T_2_ 0.9%) for DIP-MRF.

Supplementary Figure 4 demonstrates the importance of applying dropout in DIP-MRF, with simulation results shown for the 5HB/150 ms sequence. Without dropout, the nRMSE reached a minimum (T_1_ 1.7%, T_2_ 1.0%) after approximately 5,000 iterations. The nRMSE increased gradually with further training due to overfitting to noise and undersampling artifacts, reaching (T_1_ 2.2%, T_2_ 1.4%) after 30,000 iterations. Using dropout improved the reconstruction accuracy, as the minimum nRMSE was lower compared to the 0% dropout case, and it reduced overfitting, allowing the network to be trained for longer without causing the nRMSE to increase. For example, with 20% dropout, the nRMSE reached a minimum of (T_1_ 1.5%, T_2_ 0.8%) after 12,000 iterations and only increased slightly to (T_1_ 1.7%, T_2_ 1.0%) after 30,000 iterations.

### Phantom Experiments

**FIGURE 6:**
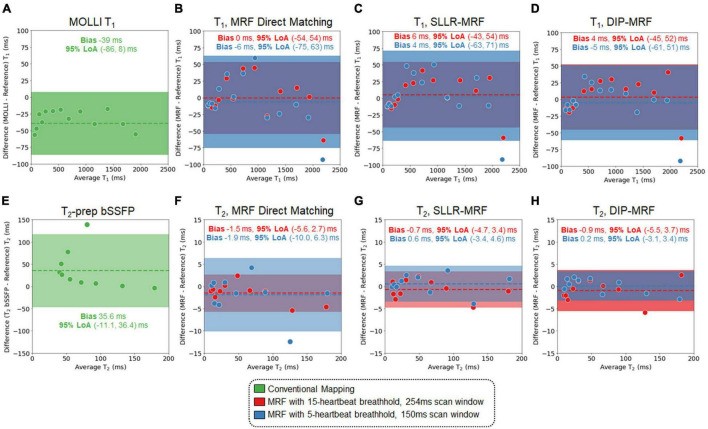
Bland-Altman plots from the phantom study. Plots are shown for T_1_ comparing **(A)** MOLLI and cardiac MRF with **(B)** direct matching, **(C)** SLLR-MRF, and **(D)** DIP-MRF reconstructions relative to gold standard measurements using an inversion recovery sequence. Similarly, plots are shown for T_2_ comparing **(E)** T_2_-prepared bSSFP and cardiac MRF with **(F)** direct matching, **(G)** SLLR-MRF, and **(H)** DIP-MRF reconstructions relative to gold standard measurements using a single-echo spin echo sequence. Results are shown for both 15HB/254 ms and 5HB/150 ms MRF sequences. The bias is indicated by a dotted line, and the 95% limits of agreement (LoA) are indicated by the solid colors.

Bland-Altman plots showing the agreement between 15HB/254 ms MRF, 5HB/150 ms MRF, and conventional mapping sequences relative to reference values are shown in Figure 6; linear regression plots of the same data are shown in Supplementary Figure 5, and T_2_ measurements in all 14 vials (including vials with T_2_ > 200 ms) are given in Supplementary Figures 6, 7. There were no significant differences in T_1_ or T_2_ relative to reference values for all MRF methods. Using DIP-MRF, the bias and 95% limits of agreement (LoA) for T_1_ were 4 ms (−45, 52)ms for the 15HB/254 ms sequence and −5 ms (−61, 51) ms for the 5HB/150 ms sequence; for T_2_, they were −0.9 ms (−5.5, 3.7) ms for the 15HB/254 ms sequence and 0.2 ms (−3.1, 3.4) ms for the 5HB/150 ms sequence. In general, DIP-MRF yielded narrower limits of agreement compared to direct matching and SLLR-MRF. MOLLI slightly underestimated T_1_ with a bias of −39 ms and 95% LoA of (−86, 8) ms. T_2_-prep bSSFP overestimated T_2_ with a bias of 35.6 ms and 95% LoA of (−45.9, 117.2) ms. This overestimation was larger for vials with short T_2_ values below approximately 100 ms, which is apparent on the linear regression plots (Supplementary Figure 5). The correlation coefficients were similar among all reconstructions for the 15HB/254 ms MRF sequence, with all R^2^> 0.998. For the 5HB/150 ms sequence, the correlation was slightly higher for DIP-MRF (R^2^= 0.999 for T_1_, R^2^= 1.000 for T_2_) compared to direct matching (R^2^= 0.998 for T_1_, R^2^= 0.995 for T_2_) and SLLR-MRF (R^2^= 0.998 for T_1_, R^2^= 0.999 for T_2_).

### Scans in Healthy Subjects

**FIGURE 7:**
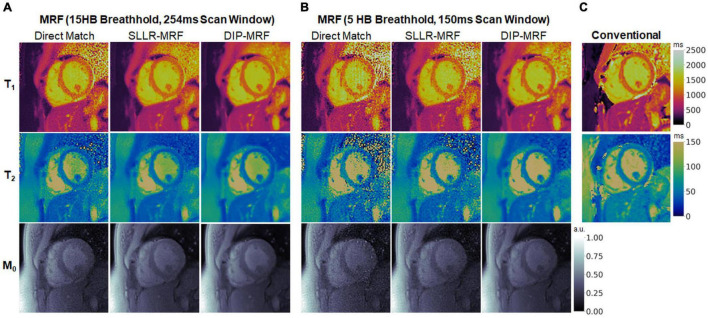
Cardiac MRF T_1_, T_2_, and M_0_ maps from a healthy subject. Maps reconstructed using direct matching, SLLR-MRF, and DIP-MRF are shown for **(A)** the 15HB/254 ms MRF sequence and **(B)** the 5HB/150 ms MRF sequence. **(C)** Conventional MOLLI and T_2_-prepared bSSFP maps are shown for comparison. All maps were cropped to a 100 × 100 region centered over the heart.

Representative maps in a healthy subject using 15HB/254 ms MRF, 5HB/150 ms MRF, and conventional mapping sequences are shown in Figure 7. Additional examples are provided in Supplementary Figures 8–10. Some noise enhancement was observed with direct matching for the 15HB/254 ms MRF sequence, with better map quality using SLLR-MRF and DIP-MRF reconstructions. The improvement using DIP-MRF was especially pronounced for the 5HB/150 ms sequence; direct matching led to severe noise enhancement and aliasing artifacts, SLLR-MRF provided only moderate noise suppression, and DIP-MRF gave the best suppression of noise and aliasing artifacts while preserving high resolution details, such as the papillary muscles.

**FIGURE 8:**
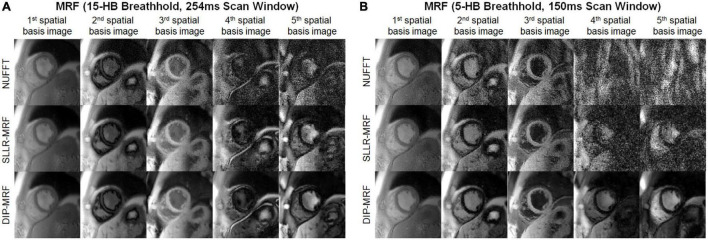
Cardiac MRF spatial basis images from a healthy subject. Spatial basis images from **(A)** the 15HB/254 ms MRF scan and **(B)** the 5HB/150 ms MRF scan are shown, reconstructed using (top row) NUFFT gridding, (middle row) SLLR-MRF, and (bottom row) DIP-MRF. Noise enhancement was observed with NUFFT gridding and to a lesser extent SLLR-MRF, while DIP-MRF yielded the best image quality. Although they tend to look similar, the contrasts of the spatial basis images in panels **(A,B)** are not expected to be identical, as a different subspace (derived from the SVD of a dictionary of signal evolutions) is calculated separately for each scan. All images were cropped to a 100 × 100 region centered over the heart.

Figure 8 shows examples of spatial basis images from DIP-MRF compared to those from conventional NUFFT gridding and SLLR-MRF. Noise enhancement was observed with NUFFT gridding, especially for the 4th and 5th basis images, which was partially reduced using SLLR-MRF, with DIP-MR yielding the best image quality.

**FIGURE 9:**
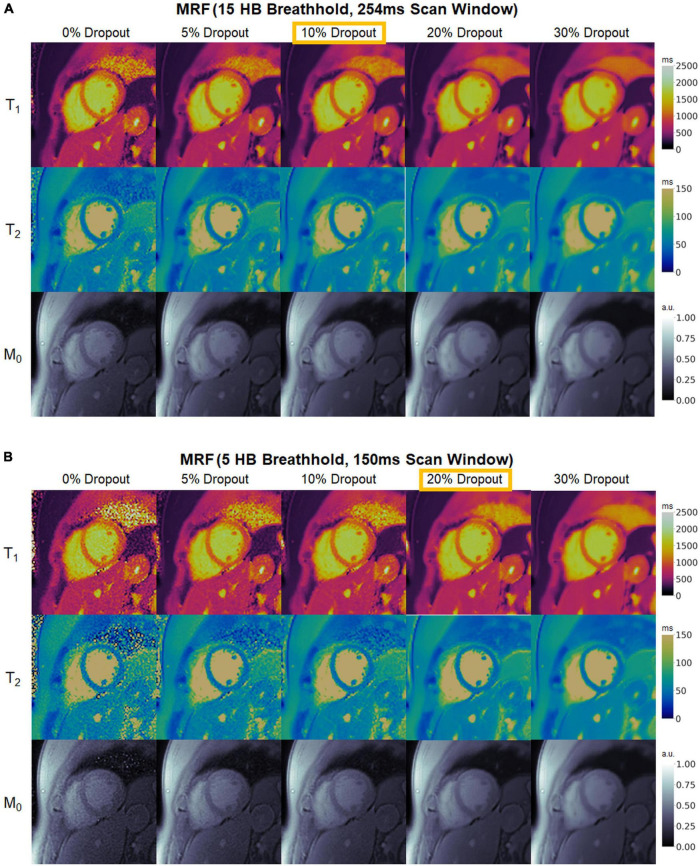
Maps from a healthy subject using DIP-MRF with different levels of dropout during training. The best dropout percentage was determined empirically to be **(A)** 10% for the 15HB/254 ms MRF sequence and **(B)** 20% for the 5HB/150 ms MRF sequence. In all cases, the number of training iterations was fixed at 30,000. Using lower dropout led to increased noise and undersampling artifacts, while higher dropout led to overly smoothed maps with a loss of high-resolution details. All maps were cropped to a 100 × 100 region centered over the heart.

Figure 9 demonstrates the effect of training DIP-MRF with different levels of dropout, akin to the simulation results in Supplementary Figure 4. From a visual inspection of the maps, the dropout level that yielded the best noise suppression while preserving high resolution details was 10% for the 15HB/254 ms sequence and 20% for the 5HB/150 ms sequence, when the number of training iterations was fixed at 30,000. Noise enhancement and residual aliasing artifacts were observed at lower dropout levels, whereas overly smoothed maps with loss of fine resolution details were seen at higher dropout levels. Results in two additional subjects are shown in Supplementary Figures 11, 12.

**FIGURE 10:**
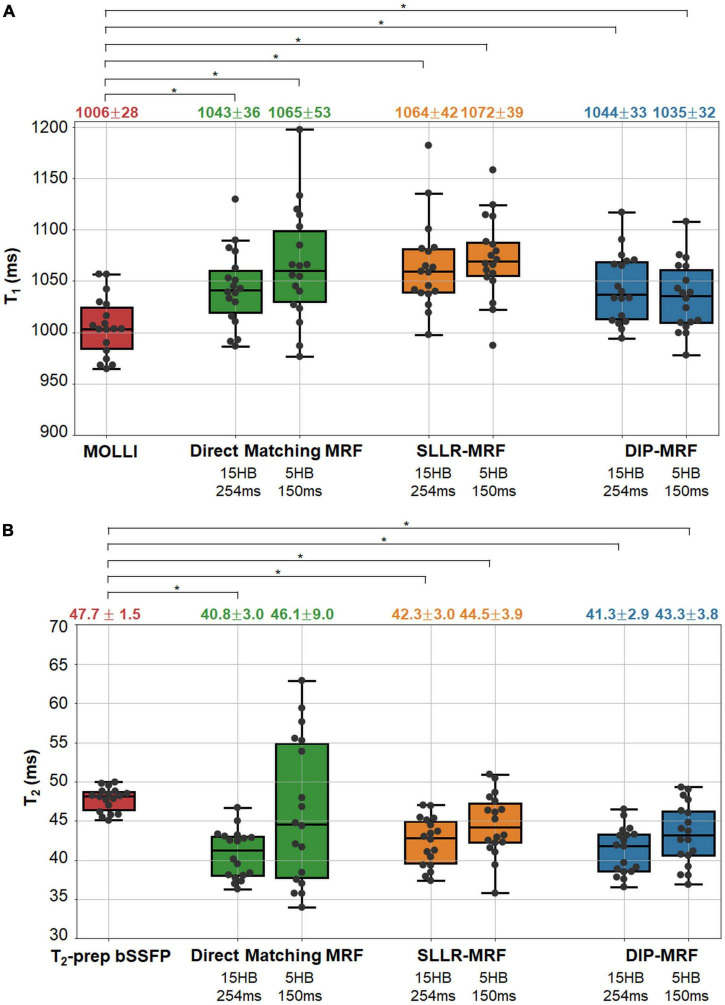
Myocardial T_1_ and T_2_ in healthy subject in the left ventricular septum. The boxplots show the distribution of mean **(A)** T_1_ and **(B)** T_2_ values using MOLLI and T_2_-prep bSSFP mapping sequences, as well as 15HB/254 ms and 5HB/150 ms MRF sequences with direct matching, SLLR-MRF, and DIP-MRF reconstructions. The top of each box indicates the upper quartile, the bottom indicates the lower quartile, and the horizontal line through the middle shows the median. The numbers above each plot indicate the mean ± standard deviation in milliseconds. Asterisks indicate a significant difference (*p* < 0.05) using a within-subjects ANOVA test with a Bonferroni *post-hoc* test for multiple comparisons.

Boxplots summarizing the average relaxation times over all subjects in the myocardial septum are shown in Figure 10. T_1_ values reported as mean ± standard deviation were: MOLLI (1,006 ± 28 ms); 15HB/254 ms MRF with direct matching (1,043 ± 36 ms), SLLR-MRF (1,064 ± 42 ms), and DIP-MRF (1,044 ± 33 ms); and 5HB/254 ms MRF with direct matching (1,065 ± 53 ms), SLLR-MRF (1,072 ± 39 ms), and DIP-MRF (1,035 ± 32 ms). T_2_ values were: T_2_-prep bSSFP (47.7 ± 1.6 ms); 15HB/254 ms MRF with direct matching (40.8 ± 3.0 ms), SLLR-MRF (42.3 ± 3.0 ms), and DIP-MRF (41.3 ± 2.9 ms); and 5HB/254 ms MRF with direct matching (46.1 ± 9.0 ms), SLLR-MRF (44.5 ± 3.9 ms), and DIP-MRF (43. ± 3.8 ms). T_1_ was significantly higher with all MRF techniques compared to MOLLI. T_2_ was significantly lower with all MRF techniques compared to T_2_-prep bSSFP, except for the 5HB/150 ms sequence with direct matching. A similar analysis of relaxation times in LV and RV blood is given in Supplementary Figure 14.

The intersubject variability, quantified as the standard deviation of the mean T_1_ or T_2_ over all subjects, was similar among all reconstructions for the 15HB/254 ms MRF scan. For the 5HB/150 ms scan, DIP-MRF yielded a lower intersubject variability (32 ms for T_1_, 3.8 ms for T_2_) compared to direct matching (53 ms for T_1_, 9.0 ms for T_2_) and SLLR-MRF (39 ms for T_1_, 3.9 ms for T_2_), although still higher than conventional mapping sequences (28 ms for T_1_, 1.5 ms for T_2_).

**FIGURE 11:**
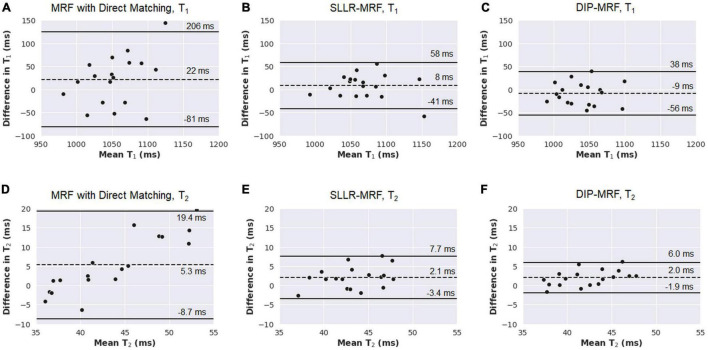
Bland-Altman plots comparing measurements from 15HB/254 ms MRF and 5HB/150 ms MRF scans with different reconstruction methods in healthy subjects. Results are shown for T_1_ using **(A)** direct matching, **(B)** SLLR-MRF, and **(C)** DIP-MRF. Similar results for T_2_ are shown in panels **(D-F)**. On each plot, the bias is indicated by a dotted line, and the 95% limits of agreement are indicated by solid lines. Note that a positive bias indicates higher T_1_ or T_2_ measurements using 5HB/150 ms MRF compared to 15HB/254 ms MRF.

Bland-Altman plots comparing relaxation times measured with 15HB/254 ms vs. 5HB/150 ms MRF scans are shown in Figure 11 (note that a positive bias indicates higher measurements using the 5HB/150 ms scan). Both scans yielded good agreement in T_1_ when using the DIP-MRF reconstruction, with a bias of −9 ms and 95% LoA (−56, 38) ms. Similar results were seen with SLLR-MRF, having a bias of 8 ms and 95% LoA (−41, 58) ms, while a larger bias (22 ms) and wider limits of agreement of (−81, 206) ms were observed with direct matching. DIP-MRF yielded the best agreement between T_2_ measurements from the 15HB/254 ms and 5HB/150 ms scans, with a bias of 2.0 ms and 95% LoA (−1.9, 6.0) ms. SLLR-MRF had a similar bias (2.1 ms) but wider limits of agreement of (−3.4, 7.7) ms. Direct matching had the largest bias (5.3 ms) and widest limits of agreement (−8.7, 19.4) ms.

**FIGURE 12:**
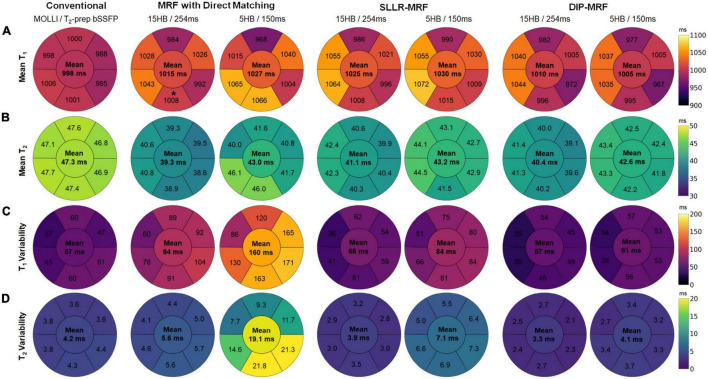
Bullseye plots showing the spatial distribution of T_1_ and T_2_ in different myocardial segments of a mid-ventricular slice in healthy subjects. **(A)** Mean T_1_ and **(B)** mean T_2_ values are shown for mid-ventricular AHA segments, with the value in the center of the bullseye indicating the average over the entire myocardium. The spatial variability (standard deviations) for T_1_ and T_2_ within each segment and over the entire myocardium are shown in panels **(C,D)**, respectively.

Figures 12A,B show the spatial distribution of T_1_ and T_2_ within individual myocardial segments and over the entire myocardium. Both 15HB/254 ms and 5HB/150 ms MRF scans showed some regional variability in T_1_ and T_2_, with higher values in the septum and lower values in the inferolateral segment. A similar but less pronounced trend was seen with MOLLI but not with T_2_-prepared bSSFP. Greater regional variability was seen with direct matching compared to SLLR-MRF and DIP-MRF.

Figures 12C,D summarize the intrasubject variability for T_1_ and T_2_, quantified as the mean of the standard deviations over all subjects, shown within each myocardial segment and over the entire myocardium. Compared to MOLLI (57 ms), the intrasubject variability in T_1_ over the entire myocardium was significantly higher using the 15HB/254 ms MRF sequence with direct matching (94 ms); this variability was reduced with SLLR-MRF (66 ms) and DIP-MRF (57 ms) and was not significantly different from MOLLI. For the 5HB/150 ms MRF sequence, the intrasubject variability was significantly higher than MOLLI when using direct matching (160 ms) and SLLR-MRF (86 ms); DIP-MRF yielded the lowest variability (61 ms) with no significant difference relative to MOLLI. Compared to T_2_-prep bSSFP (4.2 ms), the intrasubject variability in T_2_ over the entire myocardium using the 15HB/254 ms MRF sequence was significantly higher using direct matching (5.6 ms), non-significantly lower using SLLR-MRF (3.9 ms), and significantly lower using DIP-MRF (3.3 ms). For the 5HB/150 ms MRF sequence, the intrasubject variability was significantly higher than T_2_-prep using direct matching (19.1 ms) and SLLR-MRF (7.1 ms); DIP-MRF yielded the lowest variability (4.1 ms) with no significant difference relative to T_2_-prep bSSFP.

### Patient Scans

**FIGURE 13:**
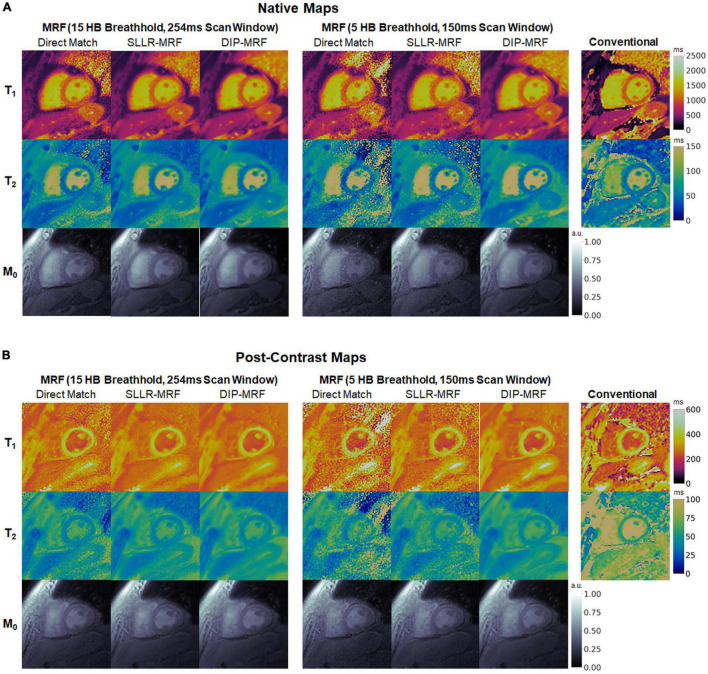
**(A)** Native and **(B)** post-contrast T_1_, T_2_, and M_0_ maps from a cardiomyopathy patient. Results are shown for conventional MOLLI and T_2_-prepared bSSFP sequences, as well as 15HB/254 ms and 5HB/150 ms MRF sequences using direct matching, SLLR-MRF, and DIP-MRF reconstructions. All maps were cropped to a 100 × 100 region centered over the heart.

Representative maps from a cardiomyopathy patient are shown in Figure 13, with additional patient examples provided in Supplementary Figures 15, 16. In both native and post-contrast maps in patients, DIP-MRF yielded the best suppression of noise and aliasing artifacts, especially for the shortened 5HB/150 ms acquisition, where direct matching led to severe noise and artifacts that were only moderately improved with the SLLR-MRF reconstruction.

**FIGURE 14:**
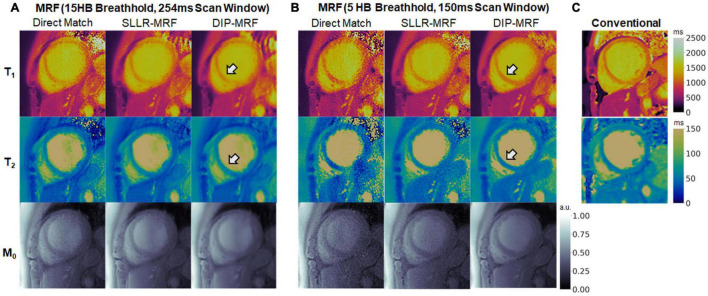
Example of reduced motion artifacts using the shortened 5HB/150 ms MRF acquisition in a cardiomyopathy patient. **(A)** The myocardial septum (white arrow) appeared blurred using the 15HB/254 ms MRF sequence due to failed breathholding and residual cardiac motion during the acquisition window. **(B)** Motion artifacts were reduced with the shortened 5HB/150 ms MRF sequence. DIP-MRF yielded improved map quality with less noise compared to the direct matching and SLLR-MRF. **(C)** MOLLI and T_2_-prepared bSSFP maps are shown for reference. All maps were cropped to a 100 × 100 region centered over the heart.

Figure 14 shows one example of a patient scan where the 15HB breathhold and 254 ms acquisition window resulted in motion artifacts. In this case, motion caused blurring of the myocardial wall and an artifactual increase in septal relaxation times due to partial volume effects between myocardium and blood, with DIP-MRF yielding T_1_ 1263 ± 48 ms and T_2_ 55.8 ± 6.5 ms. To confirm the presence of motion, a sliding window reconstruction was performed (window size = 48 TRs) to visualize one image per heartbeat, shown in Supplementary Figure 17. This analysis confirmed that the patient breathed halfway during the scan, and residual cardiac motion was apparent in the later heartbeats. Motion and partial volume effects were reduced using the shorter 5HB breathhold and 150 ms acquisition window, leading to a sharper depiction of the myocardial wall and lower septal relaxation times of T_1_ 1130 ± 27 ms and T_2_ 48.8 ± 4.1 ms (although T_1_ and T_2_ were still elevated compared to healthy subjects). Conventional MOLLI and T_2_-prep bSSFP mapping values in this patient were T_1_ = 1,122 ± 47 ms and T_2_ = 50.1 ± 4.1 ms.

**FIGURE 15:**
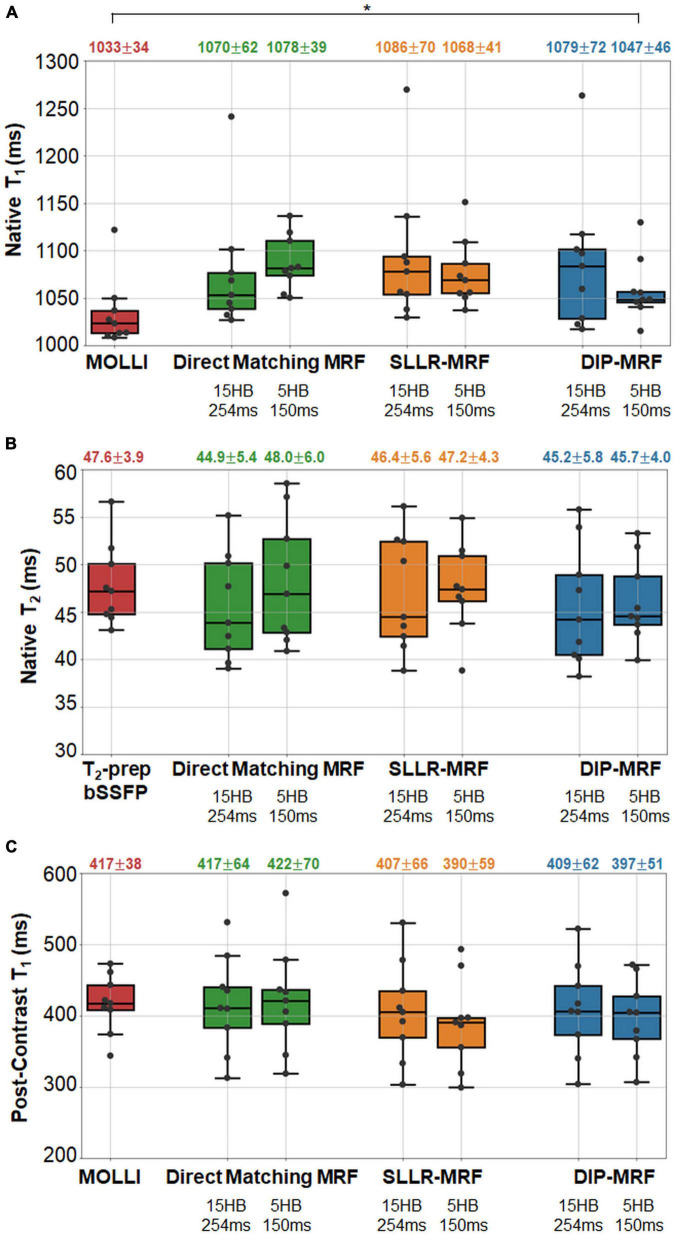
Relaxation times in the myocardial septum in cardiomyopathy patients. The boxplots summarize the **(A)** native T_1_, **(B)** native T_2_, and **(C)** post-contrast T_1_ using conventional mapping sequences, as well as 15HB/254 ms MRF and 5HB/150 ms MRF with direct matching, SLLR-MRF, and DIP-MRF reconstructions. The top of each box indicates the upper quartile, the bottom indicates the lower quartile, and the horizontal line through the middle shows the median. The numbers above each plot indicate the mean ± standard deviation over all patients. Asterisks indicate a significant difference (*p* < 0.05) using a within-subjects ANOVA test with a Bonferroni *post-hoc* test for multiple comparisons. Native mapping was performed in all ten patients. Post-contrast MRF was acquired in all ten patients, while post-contrast MOLLI was only collected in nine patients.

Boxplots summarizing the distribution of native and post-contrast relaxation times in the myocardial septum in patients are shown in Figure 15. Using the DIP-MRF reconstruction, both 15HB/254 ms MRF (1,079 ± 72 ms) and 5HB/150 ms MRF (1,047 ± 46 ms) acquisitions yielded higher native T_1_ than MOLLI (1,033 ± 34 ms); this difference was statistically significant for 5HB/150 ms DIP-MRF. Native T_2_ was non-significantly lower with both 15HB/254 ms MRF (45.2 ± 5.8 ms) and 5HB/150 ms MRF (45.7 ± 4.0 ms) compared to T_2_-prep bSSFP (47.6 ± 3.9 ms). Patients had higher native T_1_ than healthy subjects, but this trend was not significant for MOLLI, 15HB/254 ms MRF, or 5HB/150 ms MRF. Compared to healthy subjects (45.2 ms), native T_2_ in patients was significantly lower with 15HB/254 ms MRF (41.3 ms) and non-significantly lower with 5HB/150 ms MRF (43.3 ms). No difference between patients and healthy subjects was seen with T_2_-prep bSSFP (47.6 vs. 47.7 ms). There were no significant differences in post-contrast T_1_ among MOLLI (417 ± 38), 15HB/254 ms MRF (409 ± 62 ms), or 5HB/150 ms MRF (397 ± 51 ms). Post-contrast myocardial T_2_ was 37.9 ± 3.0 ms using 15HB/254 ms MRF and 38.7 ± 3.5 ms using 5HB/150 ms MRF (Supplementary Figure 18). Post-contrast T_2_ bSSFP data were only acquired in a subset of three patients; a comparison of post-contrast T_2_ bSSFP and MRF in these patients is provided in Supplementary Table 1. An analysis of native and post-contrast relaxation times in LV and RV blood in patients is given in Supplementary Figure 19.

## Discussion

This study introduced a self-supervised deep learning reconstruction for cardiac MRF, called DIP-MRF, that combines low-rank subspace modeling with the denoising capabilities of a deep image prior. DIP-MRF was shown to reduce noise and aliasing artifacts in tissue property maps compared to conventional dictionary matching and a low-rank subspace reconstruction with spatial and locally low rank constraints (SLLR-MRF). DIP-MRF was leveraged to shorten the breathhold duration of cardiac MRF from 15 to 5 heartbeats and the diastolic acquisition from 250 to 150 ms *in vivo*, which can potentially reduce motion artifacts, especially for patients who have difficulty performing long breathholds or who have elevated heart rates. By minimizing motion, the shortened acquisition may also decrease partial volume artifacts between myocardium and blood, leading to more accurate and reproducible myocardial T_1_ and T_2_ measurements. This effect was demonstrated in Figure 14, where motion resulted in an artifactual increase in myocardial T_1_ and T_2_ with the longer MRF scan that was mitigated by shortening the breathhold and scan window.

In most deep learning reconstructions, a neural network is pre-trained using a large number of reference images. For MRF, such training data would consist of “ground truth” tissue property maps (the network output) paired with a time series of undersampled images or k-space measurements (the network input). While it is possible to collect such training data in stationary organs, like the brain, it is more challenging in the heart due to physiological motion and the long scan times that would be required to collect fully-sampled MRF data (on the order of several minutes). Additionally, the fingerprints in cardiac MRF are dependent on the subject’s cardiac rhythm because the scan uses prospective ECG triggering, so many datasets would potentially be needed to ensure the network provides accurate tissue property estimates independent of a patient’s cardiac rhythm. DIP-MRF addresses these challenges by eliminating the need for prior training. Instead, training is performed *de novo* after each MRF acquisition, and the only requirements for training data are the undersampled k-space measurements from the current scan and the patient’s cardiac rhythm timings from the ECG. The self-supervised training used in DIP-MRF ensures that the reconstructed T_1_, T_2_, and M_0_ maps and spatial basis images are consistent with the acquired k-space data and with a mathematical model of the MRF signal generation and data sampling process.

One limitation of this work is the long computation time of approximately 1.1 h, since training is performed *de novo* for each scan. Nevertheless, this work used strategies to accelerate the calculation of forward model during training. The spiral k-space data were shifted onto a Cartesian grid using GROG, which allowed use FFT rather than more time-consuming NUFFT operations during training. Without GROG pre-interpolation, the DIP-MRF reconstruction took 5.3 h. A pre-trained Fingerprint Generator Network was also used in place of a Bloch equation simulation to rapidly generate fingerprints for arbitrary T_1_, T_2_, and cardiac rhythm timings. The time needed to simulate fingerprints at 192^2^ voxel locations (the matrix size used for all datasets in this work) was over 8 min using a Bloch simulation (compiled MATLAB Mex code running on 12 parallel CPUs) compared to 30 ms using the Fingerprint Generator Network on a GPU. Future work will explore ways to shorten the computation time of DIP-MRF, possibly to several minutes or less. Transfer learning may be one solution (49), where DIP-MRF is pre-trained using some *in vivo* scans, and the reconstructed maps are fine-tuned based on the acquired k-space data from the current scan.

In the original DIP publication, early stopping was used to avoid overfitting to noise, and the number of training iterations was manually tuned for each application (27). This study uses dropout to reduce overfitting (43), which allowed the network to be trained for longer and placed less dependence on manually tuning the number of iterations for early stopping. Simulation results showed that dropout improved the reconstruction accuracy and slowed the rate at which overfitting occurred (Supplementary Figure 4). An *in vivo* dataset was also reconstructed with different dropout levels, while keeping the number of training iterations fixed at 30,000 for simplicity, to empirically determine which settings yielded the best map quality. It was found that the shortened 5HB/150 ms MRF scan benefitted from higher dropout compared to the 15HB/254 ms scan (20 vs. 10% dropout).

In the absence of motion, the 15HB/254 ms and 5HB/150 ms MRF sequences were expected to yield equivalent T_1_ and T_2_ measurements. However, large differences were observed using the direct matching reconstruction, which was due to the noise enhancement and aliasing artifacts in maps using the 5HB/150 ms sequence, resulting in the wide limits of agreement in the Bland-Altman plots in Figure 11. Similar discrepancies were seen with SLLR-MRF to a lesser extent. Due to the improved quality of the maps, DIP-MRF yielded the closest agreement in T_1_ and T_2_ measured by the 15HB/254 ms and 5HB/150 ms sequences. DIP-MRF also yielded better precision *in vivo* compared to direct matching and SLLR-MRF. For T_1_, the intrasubject variability in healthy subjects was similar among MOLLI, 15HB/254 ms DIP-MRF, and 5HB/150 ms DIP-MRF. For T_2_, the intrasubject variability was lowest for 15HB/254 ms DIP-MRF, and similar between T_2_-prep bSSFP and 5HB/150 ms DIP-MRF. DIP-MRF also resulted in a lower intersubject variability for T_1_ and T_2_ compared to direct matching and SLLR-MRF.

Higher native T_1_ and lower native T_2_ were observed using MRF compared to conventional mapping sequences, which has been reported previously (50). MOLLI is known to underestimate T_1_ (51), and T_2_-prep bSSFP has been reported to overestimate T_2_ (52), which was observed in this study in the phantom experiment (Figure 6 and Supplementary Figures 5–7). The signal model in cardiac MRF accounts for slice profile imperfections and inversion pulse efficiency, which was shown to improve accuracy and lead to higher T_1_ measurements (50). Lower T_2_ values have been reported with FISP-based MRF sequences compared to standard techniques in other applications, which may be related to magnetization transfer (53), intravoxel dephasing (54), and motion sensitivity along the direction of the unbalanced gradient moment (i.e., slice direction).

Increased regional variability for T_1_ and to a lesser degree T_2_ was observed with MRF, with higher relaxation times in the septum and lower values in the inferolateral segment. Possible explanations may include susceptibility effects (especially in the inferolateral segment); partial volume artifacts between myocardium and epicardial fat, which could be improved with water-fat separation techniques like Dixon cardiac MRF (55) or MRF with rosette k-space sampling (56); and B_1_^+^ inhomogeneities, which could be addressed using B_1_^+^ correction (57, 58). Blood relaxation times were reported for completeness; however, blood flow into and out of the 2D imaging plane is not accounted for in the MRF signal simulation and likely affects the blood T_1_ and T_2_ estimates. Interestingly, higher T_1_ was measured in the LV compared to the RV with both MOLLI and cardiac MRF. Higher T_2_ was measured in the LV with T_2_-prep bSSFP, which has been reported previously (59), but slightly lower T_2_ was measured in the LV with cardiac MRF.

In summary, a DIP-MRF reconstruction that combines low-rank subspace modeling with a deep image prior was shown to reduce noise and aliasing artifacts in cardiac MRF T_1_, T_2_, and M_0_ mapping, which does not require pre-training with *in vivo* data. This method enables a shortened breathhold duration and cardiac acquisition window in cardiac MRF, which has the potential to improve scan efficiency and reduce motion artifacts. Future work will explore extensions of DIP-MRF to motion-resolved (cine) MRF (60, 61) and 3D cardiac MRF (62).

## Data Availability Statement

The raw data supporting the conclusions of this article will be made available by the authors, without undue reservation.

## Ethics Statement

The studies involving human participants were reviewed and approved by Institutional Review Boards of the University of Michigan Medical School (IRBMED). The patients/participants provided their written informed consent to participate in this study.

## Author Contributions

JH: study conception and design, deep learning reconstruction implementation, data collection and analysis, manuscript preparation, and approved the submitted version.

## Conflict of Interest

This study received funding from Siemens Healthineers (Erlangen, Germany). The funder had no involvement with any aspect of the study design, data collection, interpretation of results, or manuscript preparation. The author declares that the research was conducted in the absence of any commercial or financial relationships that could be construed as a potential conflict of interest.

## Publisher’s Note

All claims expressed in this article are solely those of the authors and do not necessarily represent those of their affiliated organizations, or those of the publisher, the editors and the reviewers. Any product that may be evaluated in this article, or claim that may be made by its manufacturer, is not guaranteed or endorsed by the publisher.
