# Supplementary material for: A Self-Supervised Deep Learning Reconstruction for Shortening the Breathhold and Acquisition Window in Cardiac Magnetic Resonance Fingerprinting
Source: Front Cardiovasc Med. 2022 Jun 23;9:928546. doi: 10.3389/fcvm.2022.928546 (PMC9260051; doi:10.3389/fcvm.2022.928546)
Supplement: Supplementary file 1 [file Data_Sheet_1.DOCX]

Supplementary Material

# Additional Details about Pre-training the Fingerprint Generator Network

The Fingerprint Generator Network (FGN) was pre-trained using one million cardiac MRF signal evolutions (fingerprints), which were obtained using a Bloch equation simulation. Fingerprints were simulated for 20,000 randomly generated cardiac rhythms. To create each cardiac rhythm, an average heart rate was randomly selected between 40 to 120 beats per minute (bpm). Random Gaussian noise was added to the RR intervals to introduce beat-to-beat variations in timing. The noise standard deviation was randomly selected as a fraction of the mean RR interval as follows: 0-10% of mean RR (50% probability), 10-20% of mean RR (30% probability), 20-50% of mean RR (10% probability), and 50-100% of mean RR (10% probability). Each heartbeat was also simulated with a 1% chance of a missed ECG trigger, which was modeled by doubling the RR interval. The tissue property values were randomly distributed as follows: T_1_ 50-2000ms (80% probability) and 2000-3000ms (20% probability), and T_2_ 5-100ms (40% probability), 100-300ms (30% probability), 300-500ms (20% probability), and 500-1000ms (10% probability). Fifty T_1_-T_2_ pairs were selected in this way for each of the 20,000 cardiac rhythms.

Slice profile imperfections and preparation pulse efficiency (for the inversions and T_2_ preps) were included in the Bloch equation simulations using an approach described previously [Hamilton JI, Jiang Y, Ma D, Lo W-C, Gulani V, Griswold M, Seiberlich N. Investigating and reducing the effects of confounding factors for robust T1 and T2 mapping with cardiac MR fingerprinting. *Magn Reson Imaging* (2018) 53:40–51. doi: 10.1016/j.mri.2018.06.018]. Briefly, slice profile effects were modeled by simulating an isochromat of 1000 spins along the slice encoding axis. For each individual spin, the nominal flip was scaled by the simulated slice profile. Preparation pulse efficiency mainly accounts for T_2_ relaxation that occurs during the inversion and T_2_ preparation pulses. In the cardiac MRF sequence used in this study, an adiabatic hyperbolic secant pulse was used for the inversion pulse. The adiabatic T_2_-preparation consisted of a BIR4 tip-down pulse, two 180$^{\circ}$ refocusing pulses, and a BIR4 tip-up pulse. Both types of preparation pulses were immediately followed by a spoiler gradient applied on the slice axis. The RF and gradient waveforms for the inversion and T_2_ preparations were simulated in the sequence programming environment (Siemens IDEA) and discretized at a time step of 50µs. These were then included in the Bloch equation simulation used to generate the MRF signal timecourses.

Generating the training set of fingerprints required 4 hours using compiled MATLAB Mex code running on 12 parallel CPU cores. Training the network required approximately 1 hour on a GPU (NVIDIA Tesla v100 16GB). A comparison of fingerprints output by the neural network and by a Bloch equation simulation are shown in Supplementary Figure 1.


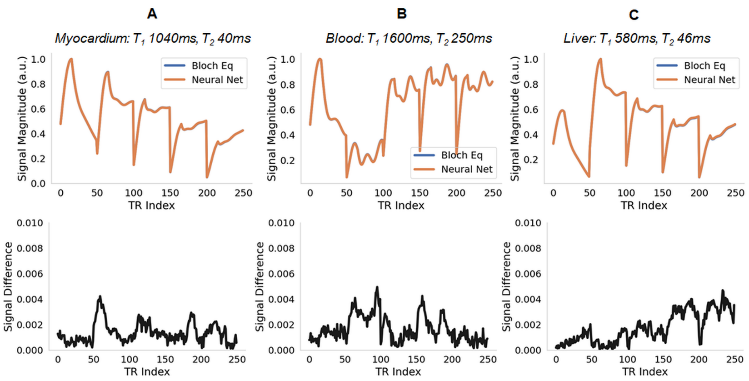


**Supplementary Figure 1**. Examples of cardiac MRF signal evolutions for three representative tissues: (A) myocardium, with T_1_=1040ms and T_2_=40ms, (B) blood with T_1_=1600ms and T_2_=250ms, and (C) liver with T_1_=580ms and T_2_=46ms. Signal evolutions are plotted from a Bloch equation simulation (blue) and using the Fingerprint Generator Network (orange) in the top row; the absolute difference between the signals is plotted in the bottom row. This example used a simulated heart rate of 70$\pm$5 beats per minute.

# Flip Angle Pattern for Shortened MRF Scans


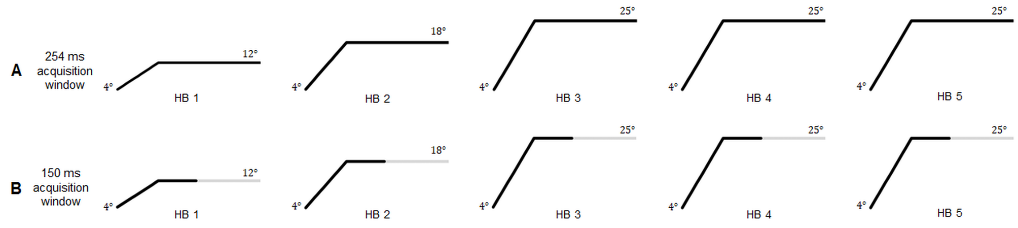


**Supplementary Figure 2**. Cardiac MRF sequences with successively shorter acquisition windows were tested, as described in the main text. The flip angle series for a sequence with a 254ms acquisition window is shown in (A). For sequences with shorter scan windows, the flip angle patten within each heartbeat was truncated to fit within the desired scan window. For example, the flip angle series corresponding to a 150ms scan window is shown in (B).

# Representative Maps from MRXCAT Simulations


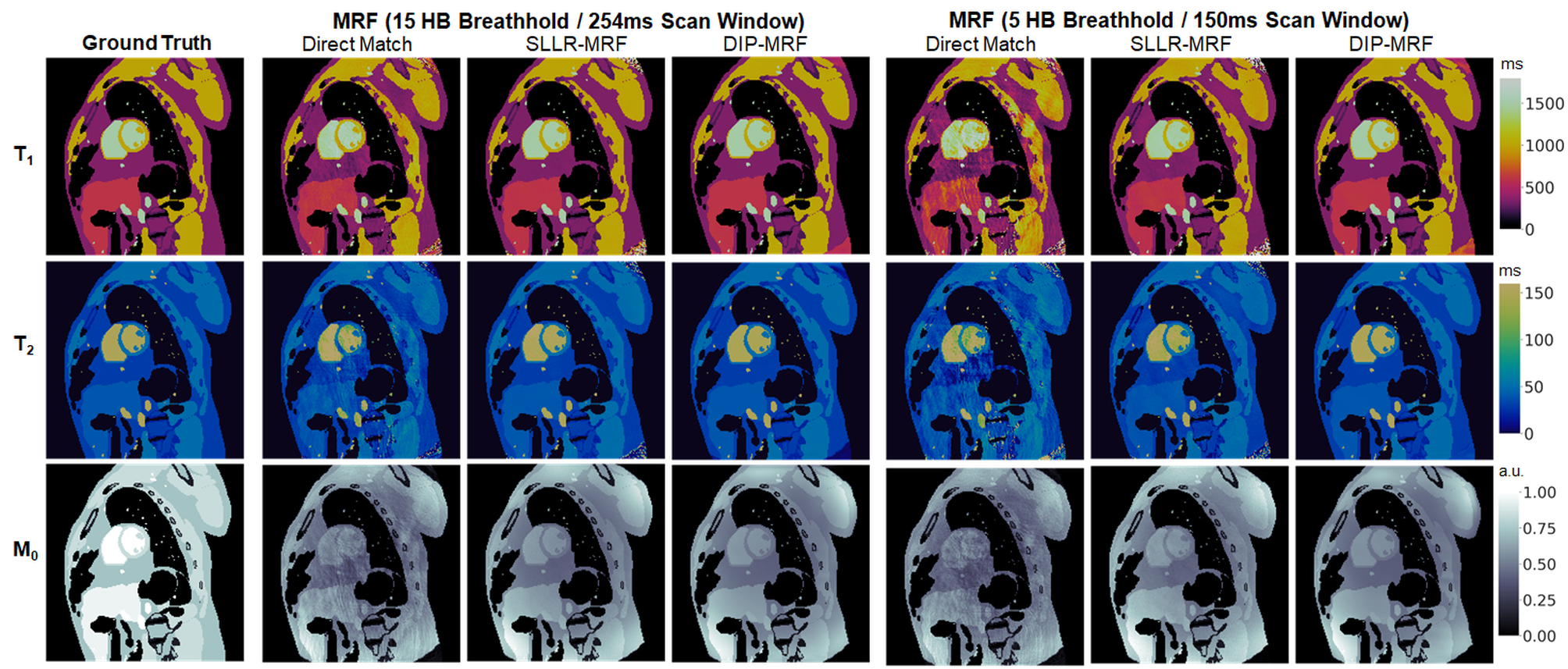


**Supplementary Figure 3**. Representative maps from the MRXCAT simulation. (A) Ground truth T_1_, T_2_, and M_0_ maps. (B) Simulated maps using the MRF sequence with a 15-heartbeat breathhold and 254m acquisition window, and (C) the MRF sequence with a 5-heartbeat breathhold and 150ms acquisition window. The MRF data were reconstructed using direct matching, SLLR-MRF, and DIP-MRF.

# Simulation Results: Dropout Regularization Improves Deep Image Prior Training


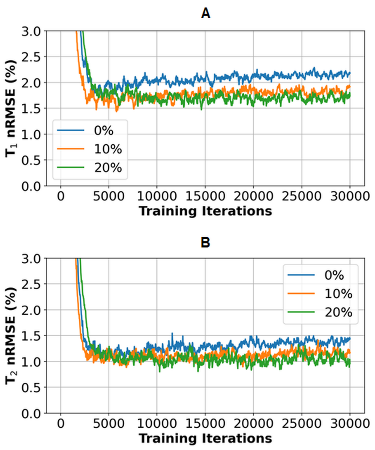


**Supplementary Figure 4**. Simulation results showing nRMSE for (A) T_1_ and (B) T_2_ as a function of training iterations, with different levels of dropout (0%, 10%, and 20%) applied when training the Image Reconstruction Network (i.e., the u-net that generates the spatial basis images). When no dropout was applied, the T_1_ and T_2_ nRMSE curves reached a minimum after approximately 5000 iterations and then began to increase slowly as the network overfit to noise and spiral undersampling artifacts. Increasing the dropout percentage improved the reconstruction performance (the minimum nRMSE was lower compared to the 0% dropout case) and made the reconstruction less prone to overfitting (when trained for longer, the nRMSE curves tended not to increase by much after reaching their minimum value).

# Additional NIST Phantom Results


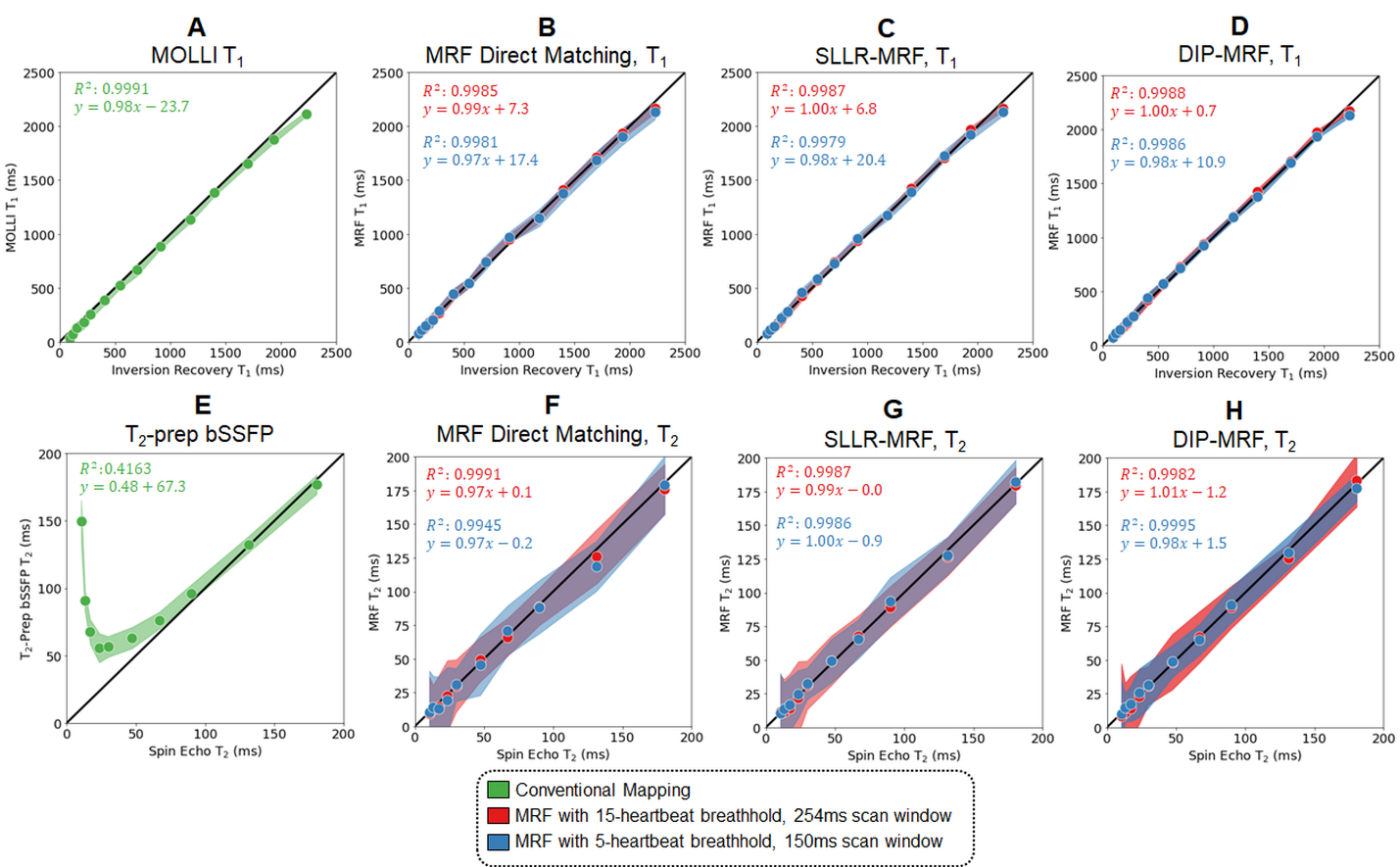


**Supplementary Figure 5**. Linear regression plots from the phantom study. Gold standard T_1_ values measured by an inversion recovery sequence were compared to (A) MOLLI, as well as the 15HB/254ms and 5HB/150ms MRF sequences with maps reconstructed using (B) direct matching, (C) SLLR-MRF, and (D) DIP-MRF. Gold standard T_2_ values measured using a single-echo spin echo sequence were compared to (E) T_2_-prepared bSSFP, as well as the 15HB/254ms and 5HB/150ms MRF sequences with maps reconstructed using (F) direct matching, (G) SLLR-MRF, and (H) DIP-MRF. Note that vials with T_2_>200ms were excluded from analysis. Each data point represents the mean T_1_ or T_2_ measured within a phantom vial. The width of each colored area indicates the standard deviation. Correlation coefficient (R^2^) and best-fit lines are also reported. MOLLI showed a slight underestimation in T_1_ (better seen on the Bland-Altman plots in Figure 6), while T_2_-prep bSSFP overestimated T_2_, especially for vials with short T_2_. All MRF variants showed good agreement with reference values, with the least variability (smallest standard deviation) and strongest correlation (highest R^2^) using the DIP-MRF reconstruction.


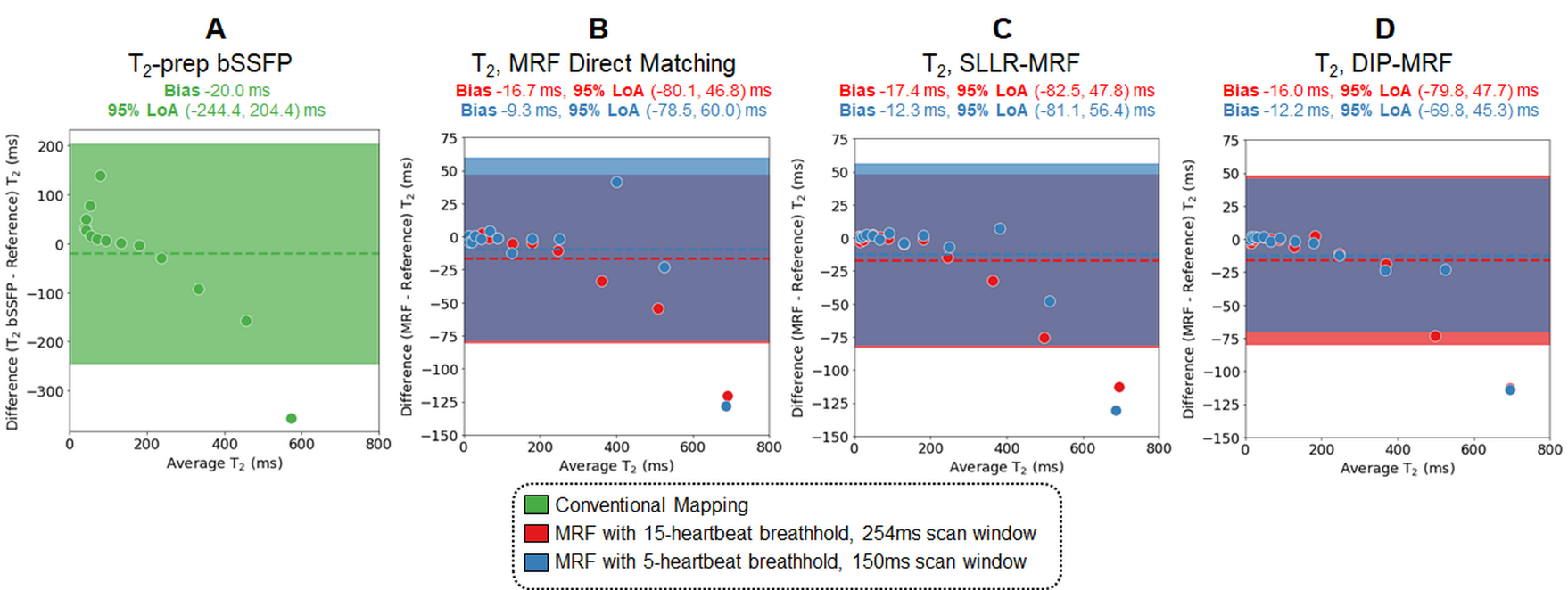


**Supplementary Figure 6.** Bland-Altman plots for T_2_ from the phantom study reported in all 14 vials. Plots are shown comparing (A) T_2_-prepared bSSFP and cardiac MRF with (B) direct matching, (C) SLLR-MRF, and (D) DIP-MRF reconstructions relative to gold standard measurements using a single-echo spin echo sequence. Results are shown for both 15HB/254ms and 5HB/150ms MRF sequences. The bias is indicated by a dotted line, and the 95% limits of agreement (LoA) are indicated by the solid colors.


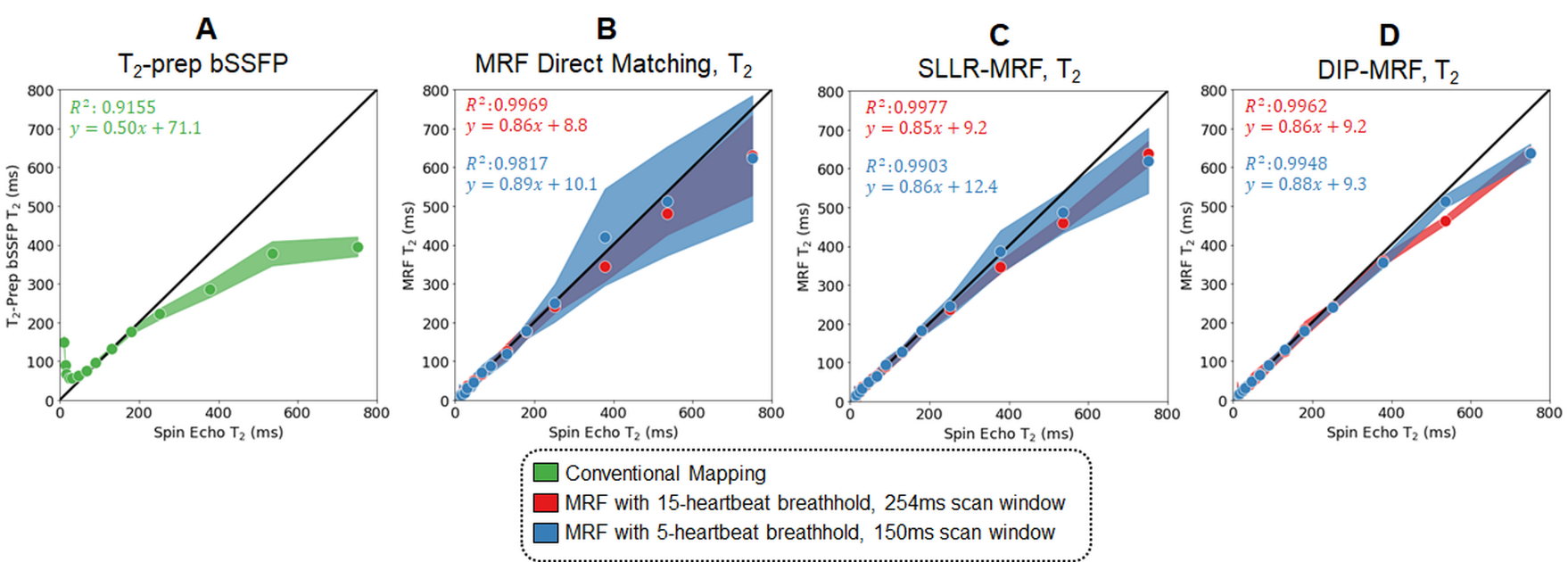


**Supplementary Figure 7**. Linear regression plots from the phantom study for T_2_ in all 14 vials. Gold standard T_2_ values measured using a single-echo spin echo sequence were compared to (A) T_2_-prepared bSSFP, as well as the 15HB/254ms and 5HB/150ms MRF sequences with maps reconstructed using (B) direct matching, (C) SLLR-MRF, and (D) DIP-MRF. Each data point represents the mean T_1_ or T_2_ measured within a phantom vial. The width of each colored area indicates the standard deviation. Correlation coefficient (R^2^) and best-fit lines are also reported.

# Examples in Additional Healthy Subjects


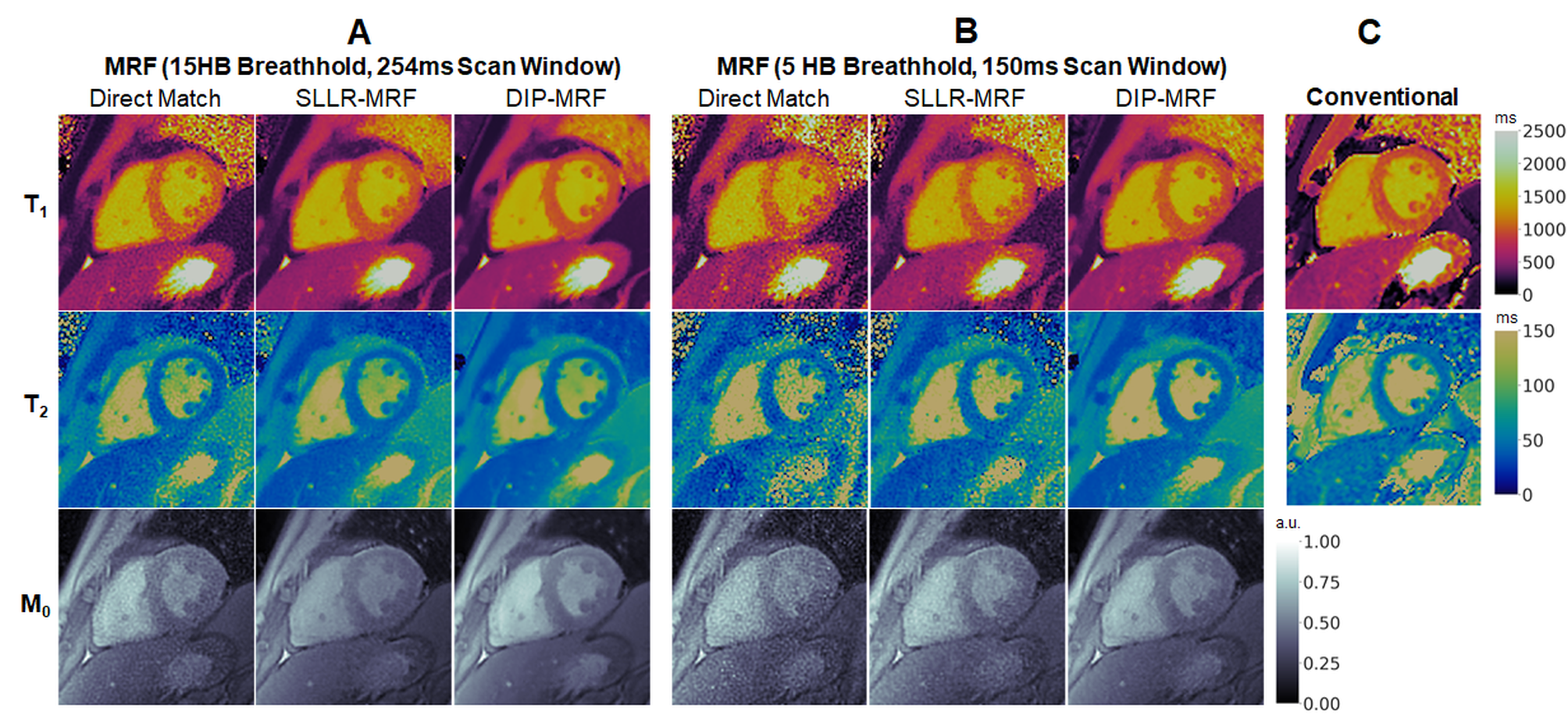


**Supplementary Figure 8**. Representative T_1_, T_2_, and M_0_ maps from an additional healthy subject using (A) MRF with a 15-heartbeat breathhold and 254ms acquisition window, and (B) MRF with a 5-heartbeat breathhold and 150ms acquisition window. The MRF maps were reconstructed using direct matching, SLLR-MRF, and DIP-MRF techniques. (C) Conventional MOLLI and T_2_-prep bSSFP maps are shown for comparison. All maps were cropped to a 100x100 region centered over the heart.


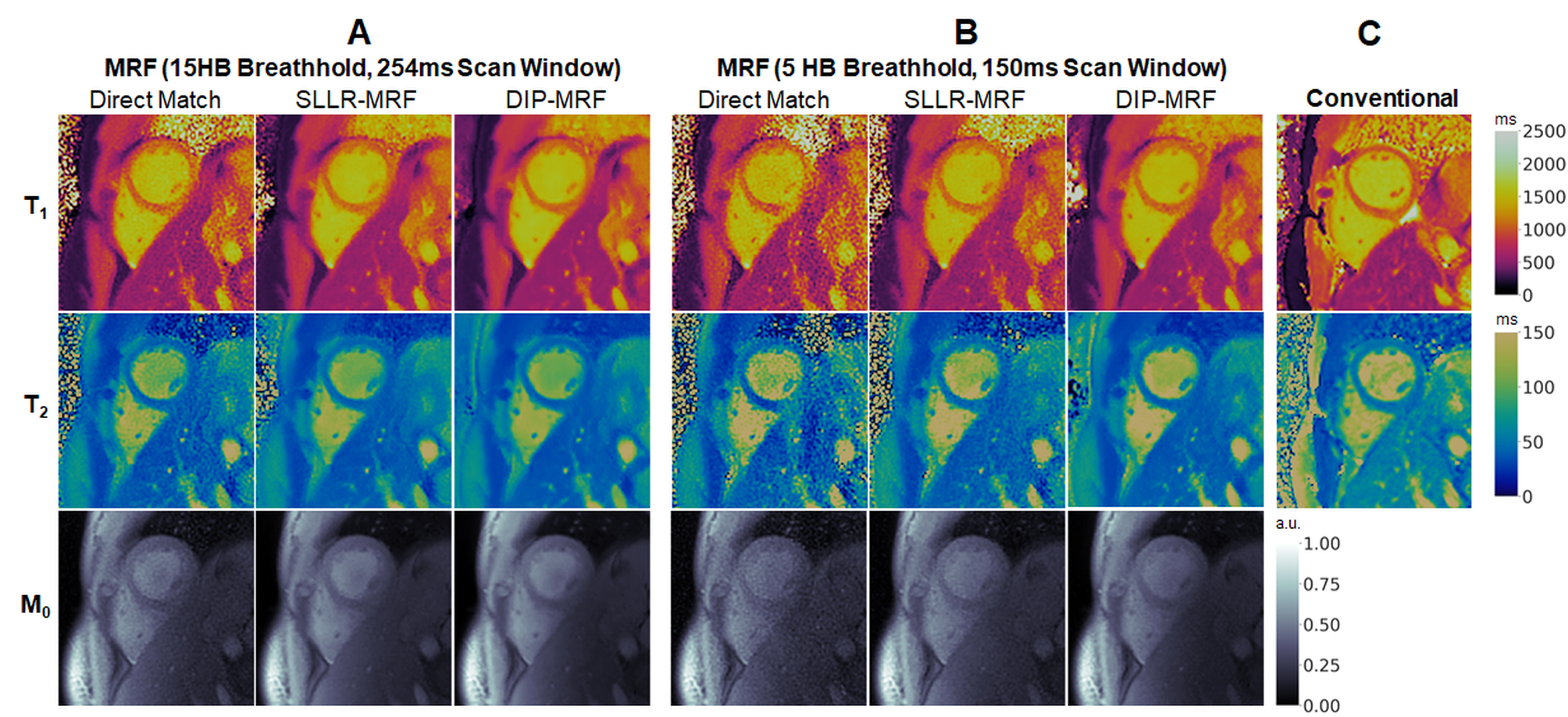


**Supplementary Figure 9**. Representative T_1_, T_2_, and M_0_ maps from an additional healthy subject using (A) MRF with a 15-heartbeat breathhold and 254ms acquisition window, and (B) MRF with a 5-heartbeat breathhold and 150ms acquisition window. The MRF maps were reconstructed using direct matching, SLLR-MRF, and DIP-MRF techniques. (C) Conventional MOLLI and T_2_-prep bSSFP maps are shown for comparison. All maps were cropped to a 100x100 region centered over the heart.


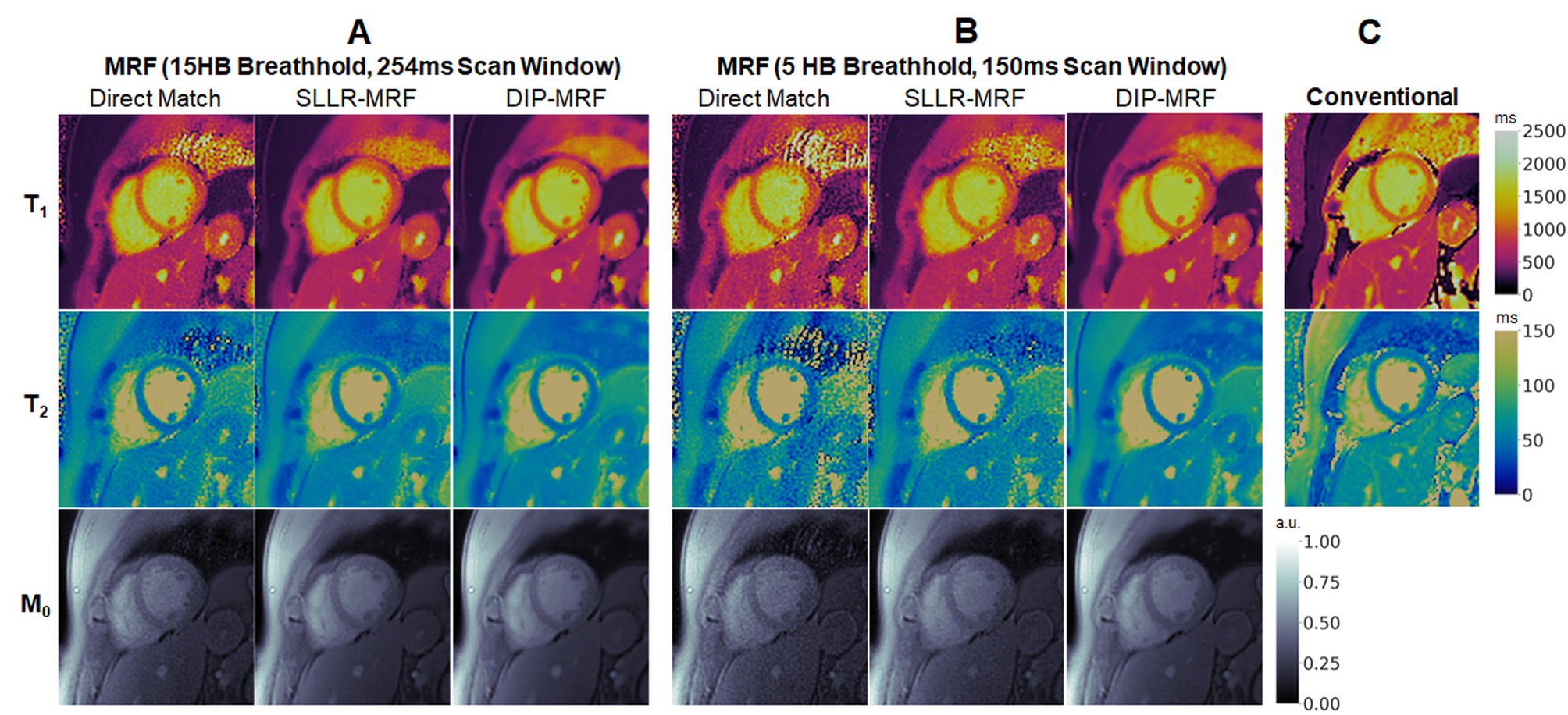


**Supplementary Figure 10**. Representative T_1_, T_2_, and M_0_ maps from an additional healthy subject using (A) MRF with a 15-heartbeat breathhold and 254ms acquisition window, and (B) MRF with a 5-heartbeat breathhold and 150ms acquisition window. The MRF maps were reconstructed using direct matching, SLLR-MRF, and DIP-MRF techniques. (C) Conventional MOLLI and T_2_-prep bSSFP maps are shown for comparison. All maps were cropped to a 100x100 region centered over the heart.

# Effect of Dropout Regularization: Examples in Additional Subjects


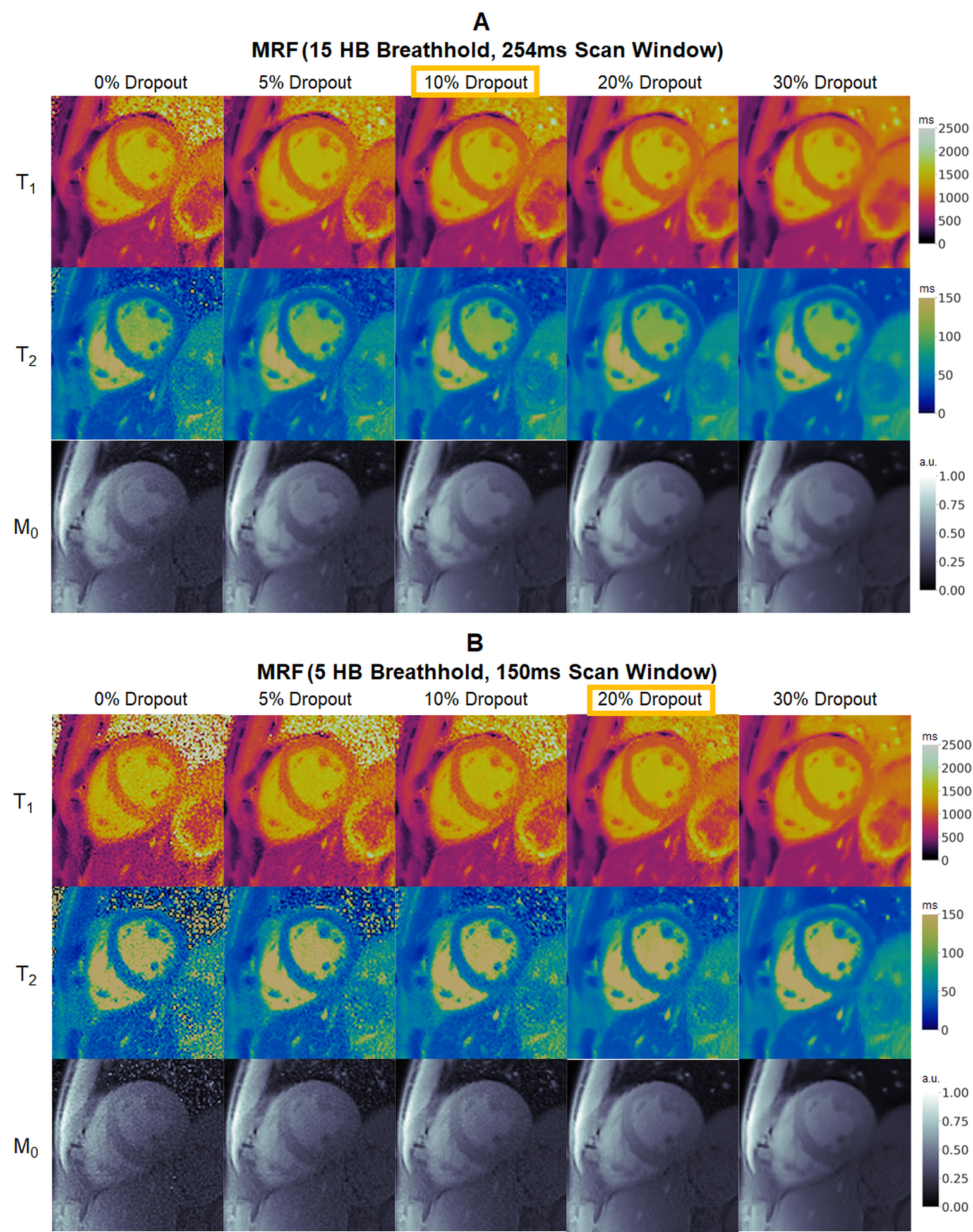


**Supplementary Figure 11**. Maps from a healthy subject using DIP-MRF with different levels of dropout during training. The best dropout percentage was determined empirically to be (A) 10% for the 15HB/254ms MRF sequence and (B) 20% for the 5HB/150ms MRF sequence. In all cases, the number of training iterations was fixed at 30,000. All maps were cropped to a 100x100 region centered over the heart.


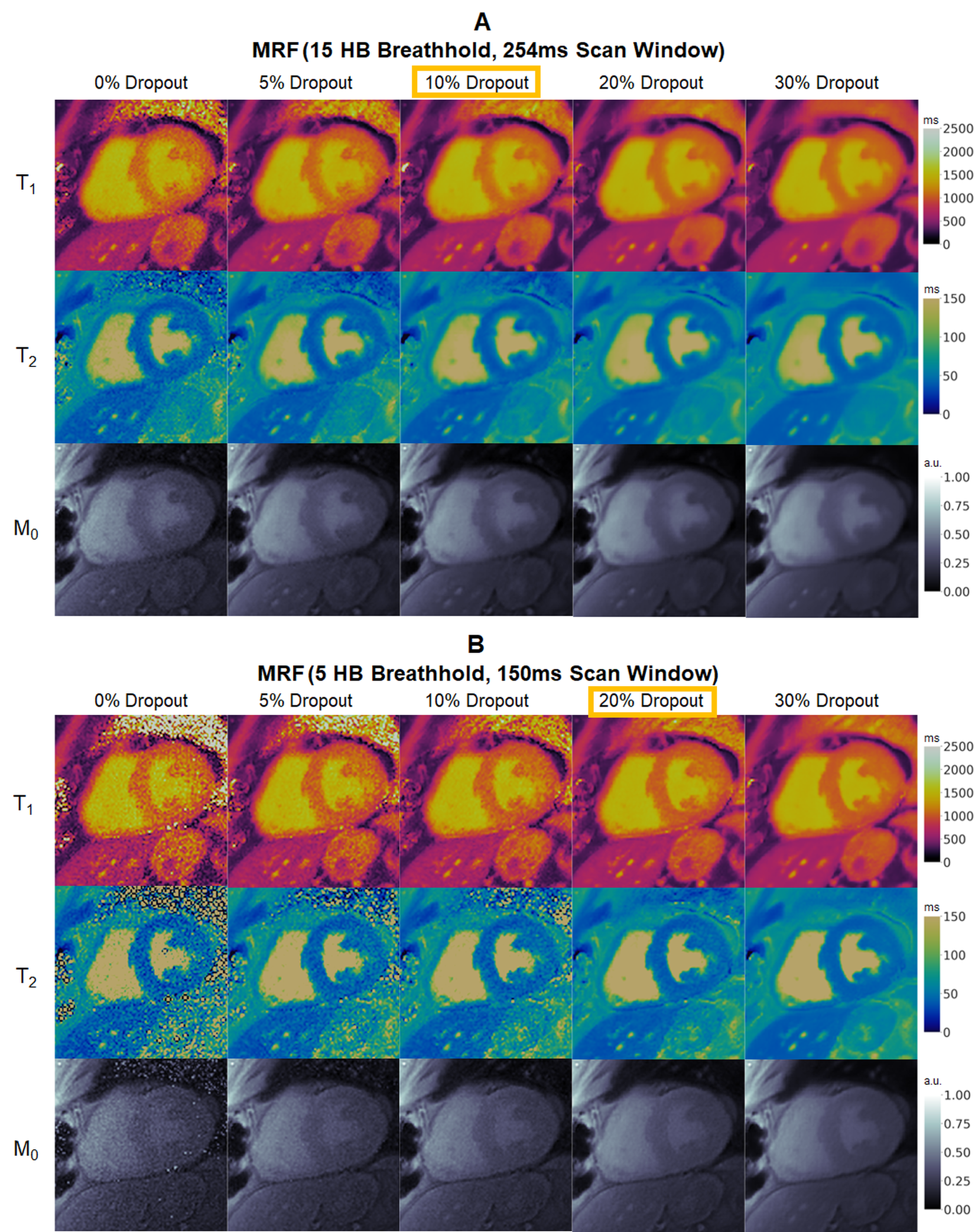


**Supplementary Figure 12**. Maps from a cardiomyopathy patient using DIP-MRF with different levels of dropout during training. The best dropout percentage was determined empirically to be (A) 10% for the 15HB/254ms MRF sequence and (B) 20% for the 5HB/150ms MRF sequence. In all cases, the number of training iterations was fixed at 30,000. All maps were cropped to a 100x100 region centered over the heart.

# Training Iterations and Dropout Level


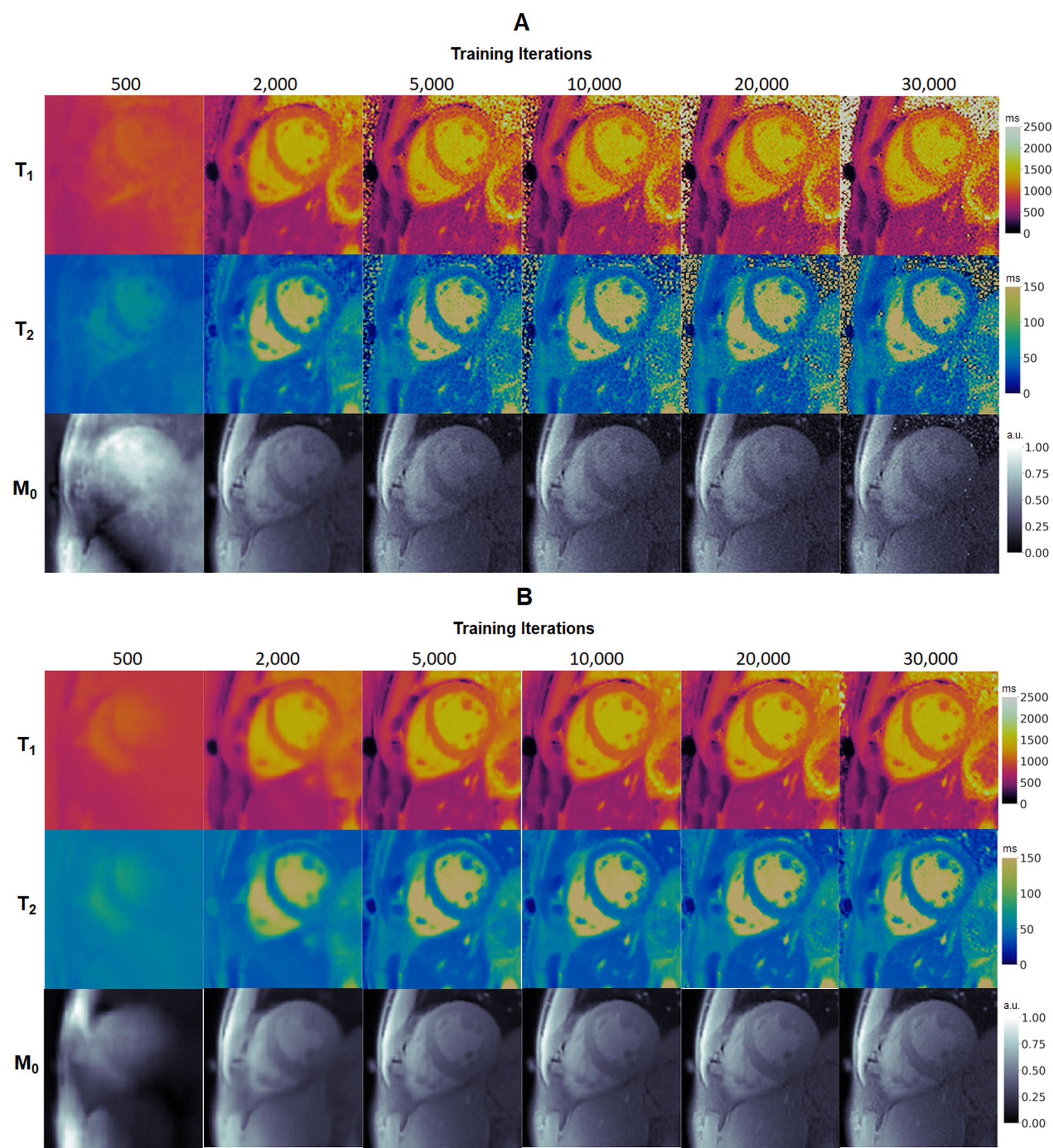


**Supplementary Figure 13.** Maps from one healthy volunteer (5HB/150ms MRF) were reconstructed using DIP-MRF with different numbers of training iterations. Training was performed (A) without dropout and (B) with 20% dropout after each convolutional layer. Without dropout, the number of training iterations has a large influence on the map quality, with noise enhancement becoming pronounced after 5000 iterations. Using dropout reduces the risk of overfitting, allowing the network to be trained for longer without having to precisely tune the number of iterations, and it leads to improved map quality with reduced noise enhancement and aliasing artifacts. This example also illustrates that the deep image prior recovers lower spatial frequency information before higher spatial frequencies, as using too few iterations results in blurred maps.

# Native Relaxation Times in LV/RV Blood in Healthy Subjects

ROIs were manually drawn in the LV and RV blood pools on native T_1_ and T_2_ maps, taking care to avoid trabeculations and the papillary muscles (Supplementary Figure 14). Differences in T_1_ and T_2_ among different methods within the same subject were assessed using a within-subjects ANOVA with a Bonferroni correction for multiple comparisons. LV T_1_ was significantly lower for both 15HB/254ms and 5HB/150ms MRF compared to MOLLI. RV T_1_ was significantly lower for 5HB/150ms MRF compared to MOLLI; no difference relative to MOLLI was observed for 15HB/254ms MRF. LV T_2_ was significantly lower for both 15HB/254ms and 5HB/150ms MRF compared to T_2_-prep bSSFP. RV T_2_ was significantly higher for 5HB/150ms MRF compared to T_2_-prep bSSFP; no difference relative to T_2_-prep bSSFP was observed for 15HB/254ms MRF. LV blood had a significantly higher T_1_ than RV blood using MOLLI and 5HB/150ms MRF; the same trend was seen with 15HB/254ms MRF but was not statistically significant. LV blood T_2_ was significantly lower with 15HB/254ms MRF compared to both T_2_-prep bSSFP and 5HB/150ms MRF. RV blood T_2_ was similar between T_2_-prep bSSFP and 15HB/254ms MRF, but significantly higher using 5HB/150ms MRF. Significantly higher T_2_ was measured in the LV versus RV blood using T_2_-prep bSSFP, which has been reported in previous studies (Emrich T, et al. *J Magn Reson Imaging*. 2022;55(5):1452-1458). The opposite trend was seen using MRF, with lower T_2_ in the LV versus RV, which was statistically significant for the 15HB/254ms scan.

While these values are reported here for completeness, the signal model used in cardiac MRF does not account for the effects of blood flowing into and out of the 2D imaging slice. Therefore, this study does not claim to measure accurate blood relaxation times using cardiac MRF.


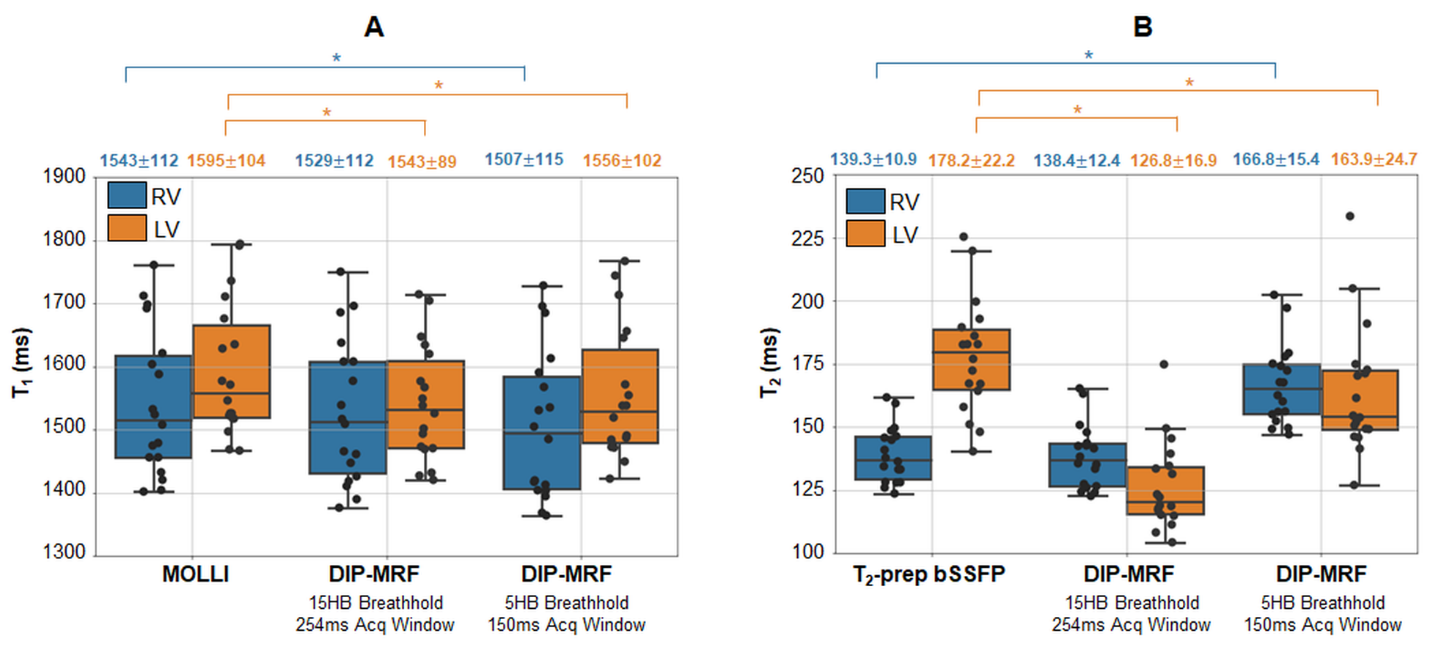


**Supplementary Figure 14.** Boxplots summarizing average (A) T_1_ and (B) T_2_ in the left ventricular (orange) and right ventricular (blue) blood pools in the healthy subject cohort. The numbers above each plot indicate the mean ± standard deviation over all subjects. The top of each box indicates the upper quartile, the bottom indicates the lower quartile, and the horizontal line through the middle shows the median

# Examples in Additional Cardiomyopathy Patients


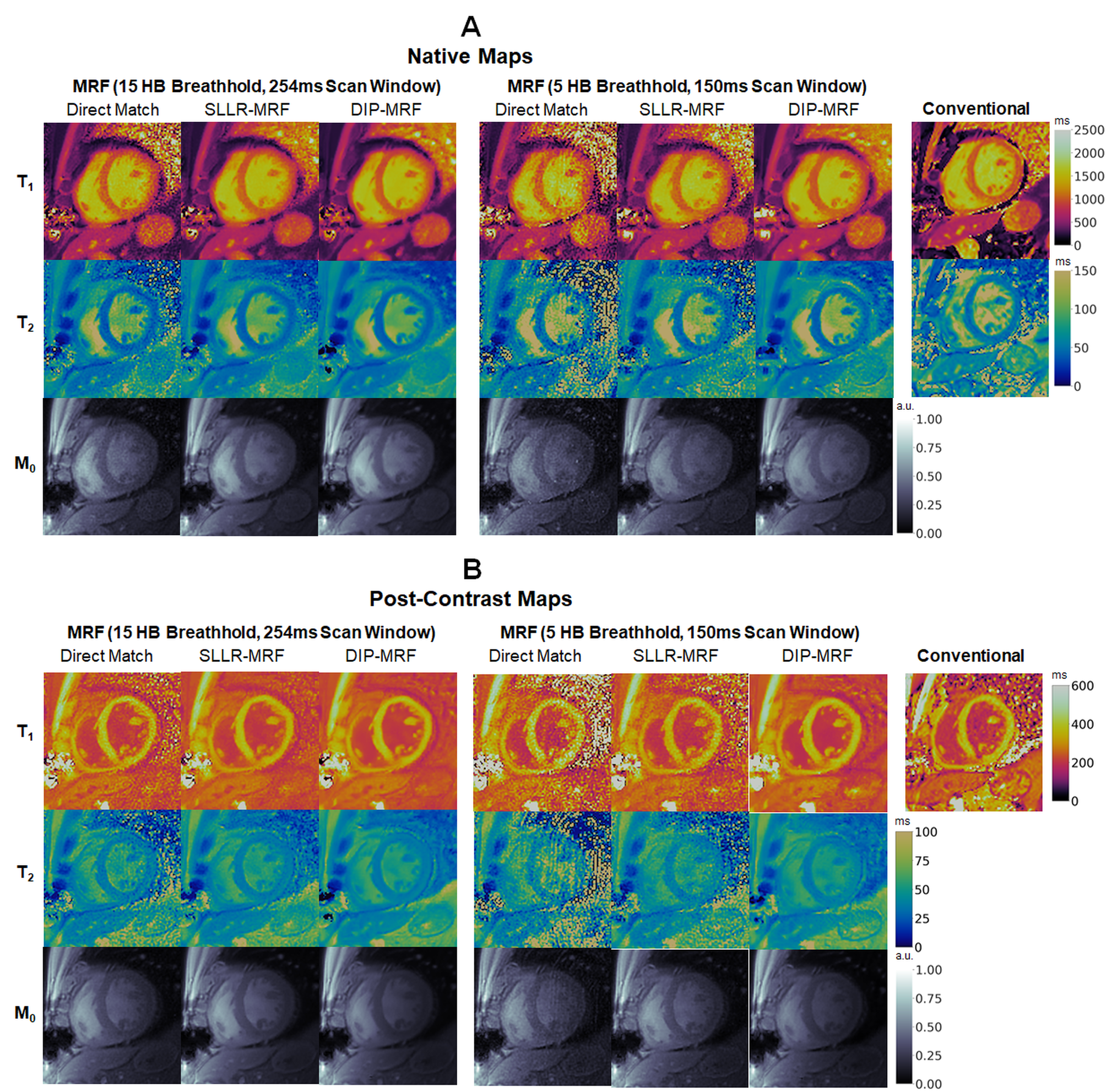


**Supplementary Figure 15**. (A) Native and (B) post-contrast maps from an additional cardiomyopathy patient. Results are shown for MRF with a 15-heartbeat breathhold and 254ms acquisition window, and for MRF with a 5-heartbeat breathhold and 150ms acquisition window, with maps reconstructed using direct matching, SLLR-MRF, and DIP-MRF. Conventional MOLLI and T_2_-prep bSSFP maps are shown for reference. No post-contrast T_2_-prep bSSFP scans were available for this patient. All maps were cropped to a 100x100 region centered over the heart.


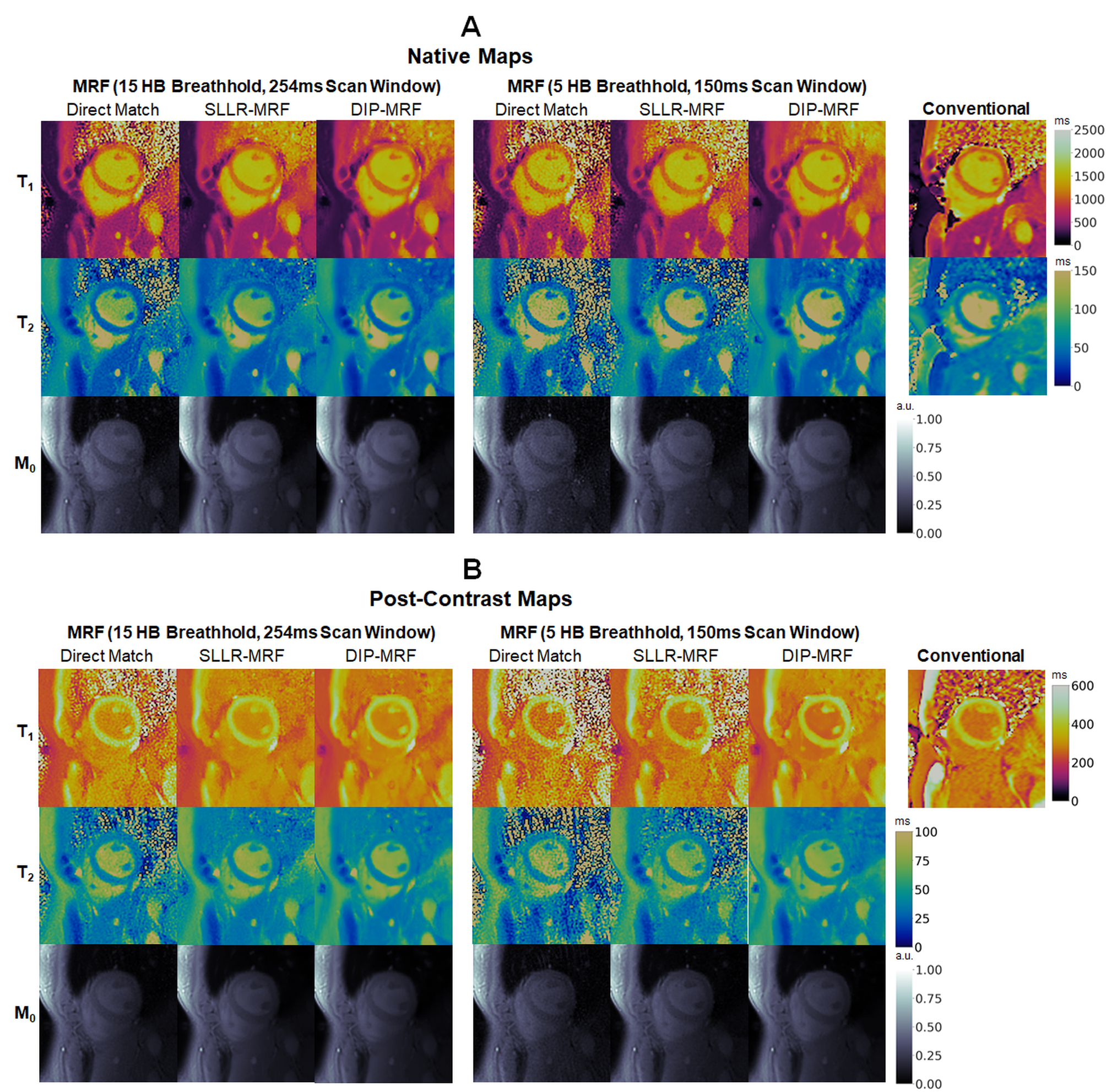


**Supplementary Figure 16**. (A) Native and (B) post-contrast maps from an additional cardiomyopathy patient. Results are shown for MRF with a 15-heartbeat breathhold and 254ms acquisition window, and for MRF with a 5-heartbeat breathhold and 150ms acquisition window, with maps reconstructed using direct matching, SLLR-MRF, and DIP-MRF. Conventional MOLLI and T_2_-prep bSSFP maps are shown for reference. No post-contrast T_2_-prep bSSFP scans were available for this patient. All maps were cropped to a 100x100 region centered over the heart.

# Demonstration of Respiratory/Cardiac Motion in a Patient Scan


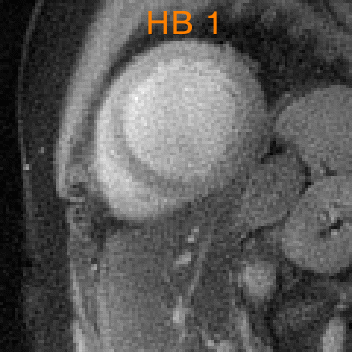


**Supplementary Figure 17**. Animated GIF demonstrating motion artifacts during an MRF scan with a 15-heartbeat breathhold and 254ms acquisition window in a patient; the corresponding tissue property maps are shown in Figure 14 in the main text. The movie shows images from a sliding window reconstruction (window size = 47 TRs), with one image reconstructed per heartbeat. The patient began to breathe after heartbeat #7, and residual cardiac motion can be seen during the later heartbeats.

# Post-Contrast Myocardial T_2_ in Patients

**Supplementary Figure 18**. Post-contrast T_2_ measured in the myocardial septum in ten cardiomyopathy patients using 15HB/254ms and 5HB/150ms MRF acquisitions reconstructed with direct matching, SLLR-MRF, and DIP-MRF. The numbers above each plot indicate the mean $\pm$ standard deviation over all patients. The top of each box indicates the upper quartile, the bottom indicates the lower quartile, and the horizontal line at the middle of the box shows the median.

**Supplementary Table 1**. Post-contrast T_2_-prep bSSFP mapping was only performed in three out of ten patients. This table summarizes the native and post-contrast T_2_ measured in the myocardial septum in these patients using T_2_-prep bSSFP, 15HB/254ms DIP-MRF, and 5HB/150ms DIP-MRF. While MRF shows the expected result that post-contrast T_2_ was slightly lower than native T_2_, the opposite trend was seen with T_2_-prep bSSFP. This may be related to the small sample size, as well as the trend of T_2_-prep bSSFP to overestimate T_2_ in species that have shorter T_1_ and T_2_ times, which was observed in the NIST phantom results (Figure 6 and Supplementary Figures 5-7).

# Relaxation Times in Blood in Patients


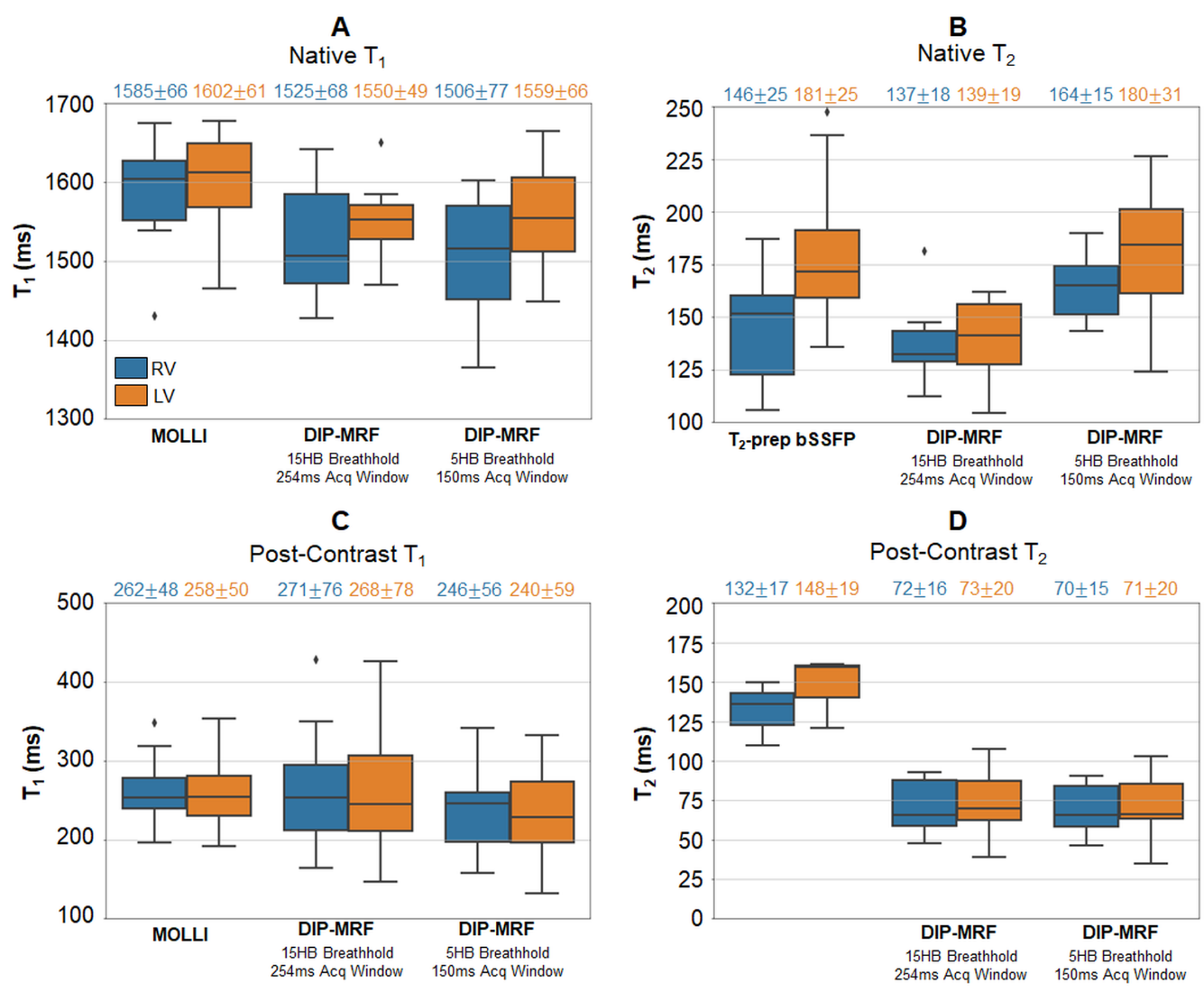


**Supplementary Figure 19**. Left and right ventricular blood relaxation times in the patient cohort, showing (A) native T_1_, (B) native T_2_, (C) post-contrast T_1_, and (D) post-contrast T_2_ measured with conventional mapping, 15HB/254ms MRF, and 5HB/150ms MRF sequences using the DIP-MRF reconstruction. The native relaxation times agreed with values measured in healthy subjects (shown in Supplementary Figure 14), with the one exception being for RV T_1_ using MOLLI, which was slightly higher in patients (1585$\pm$66ms) compared to healthy subjects (1543$\pm$112ms). Although blood relaxation times in patients are reported for completeness, the signal model in cardiac MRF does not account for the effects of blood flowing into and out of the 2D imaging slice. Therefore, this study does not claim to measure accurate blood relaxation times using cardiac MRF.

# Network Architecture: Number of Skip Connections in Image Reconstruction Network

Experiments were performed to evaluate how the number of skip connections in the u-net (the Image Reconstruction Network) impacted the reconstructed MRF maps. First, simulations were performed with the MRXCAT phantom using the 5HB/150ms MRF sequence, with complex Gaussian noise added to the k-space data having a standard deviation of 0.3% relative to the maximum amplitude of the direct current (DC) signal. Skip connections were implemented at each level of the u-net as a convolutional layer with a 1x1 kernel size, which was then concatenated to the decoder, as shown in Figure 3. The number of feature maps in the skip connection layers were varied from 4, 8, 16, 32, 64, and 128. Note that the experiments described in the main text employed 4 feature maps for the skip connections; this is the architecture shown in Figure 3. Additionally, experiments were performed using a u-net without any skip connections. The DIP-MRF reconstruction was performed using 30,000 training iterations and a dropout rate of 20%. The nRMSE was computed in the T_1_ and T_2_ maps and averaged over all non-background pixels (where the ground truth M_0_ was greater than zero). Supplementary Table 2 shows the nRMSE values. In simulations, the number of skip connections was not found to have a large impact on the reconstructed maps, with all networks yielding similar nRMSE values of around 1.6-1.7% for T_1_ and 0.8-1.0% for T_2_.

Similar experiments were performed using a 5HB/150ms MRF dataset acquired in a healthy subject. As shown in Supplementary Figure 20, using no skip connections resulted in a loss of high-resolution details. Using skip connections with a small number of feature maps (e.g., 4 or 16) yielded a good balance between noise suppression and retention of high-resolution details. Using skip connections with more feature maps (e.g., 128) resulted in noise enhancement. It may be possible to obtain better results by tuning the number of training iterations for specific network architectures, but this was not investigated here.


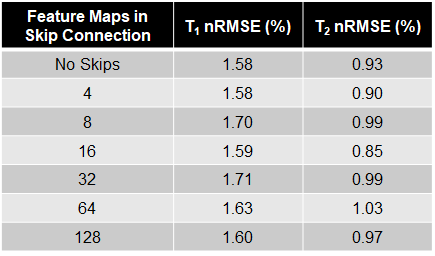


**Supplementary Table 2**. T_1_ and T_2_ nRMSE from the simulation study, where the DIP-MRF reconstruction was performed with different numbers of feature maps in the skip connection layers.


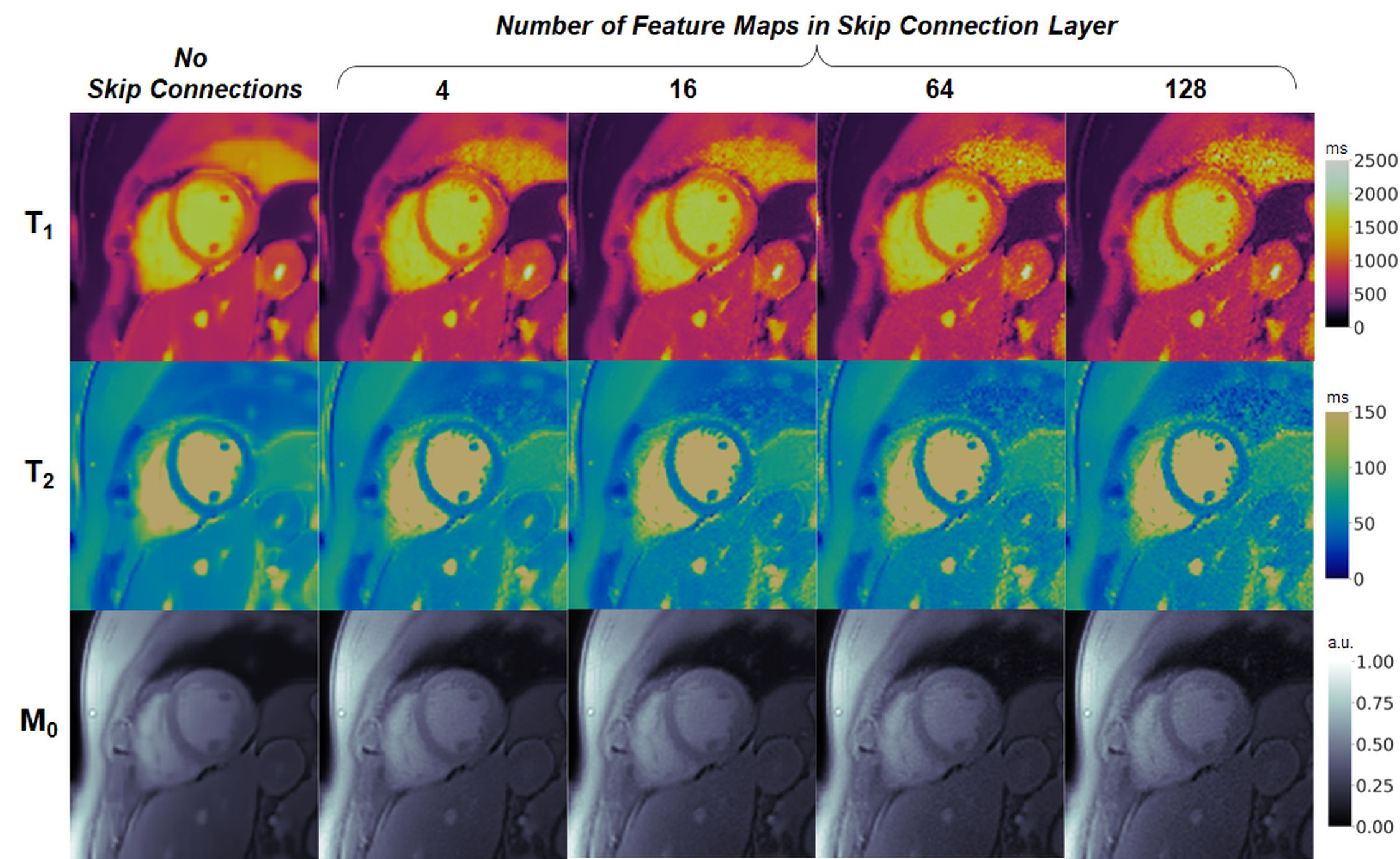


**Supplementary Figure 20.** T_1_, T_2_, and M_0_ maps from a healthy subject using an MRF sequence with a 5-heartbeat breathhold and 150ms acquisition window. The DIP-MRF reconstruction was repeated using different numbers of feature maps in the skip connection layers in the Image Reconstruction Network.
